# Supplementary material for: Prevalence of depression and associated factors among pregnant women attending antenatal care in public health institutions of Awabale Woreda, East Gojjam Zone, Northwestern Ethiopia: A cross-sectional study
Source: PLoS One. 2022 Oct 13;17(10):e0271876. doi: 10.1371/journal.pone.0271876 (PMC9560138; doi:10.1371/journal.pone.0271876)
Supplement: S1 Questionnaire — (DOCX) [file pone.0271876.s001.docx]

Annex

English version questionnaire

Instruction: This questioner has seven parts. To complete this questionnaire it takes 20 min and tries to respond to all questions. Thank you for your patience.

| SECTION ONE: SOCIO-DEMOGRAPHIC QUESTIONS | | | | | | | | | |
| --- | --- | --- | --- | --- | --- | --- | --- | --- | --- |
| Q.NO. | | Questions | | Alternative responses | | | Skip | | Code |
| Q101 | | How old are you? (completed in years) | | __________Age in years | | |  | |  |
| Q102 | | What is your Religion? | | 1. Orthodox 2. Muslim 3. Protestant 4. Catholic 5. Others | | |  | |  |
| Q103 | | What is your Ethnicity? | | 1. Amhara 2. Oromo 3. Tigre 4. Other | | |  | |  |
| Q104 | | What is current marital status? | | 1. Single 2. Married & living together 3. Married & not living together 4. Divorced 5. Widowed | | |  | |  |
| Q105 | | What is your educational status? | | 1. Unable to read and write 2. Able to read and write 3. Elementary school(grade 1-8) 4. High school(grade 9-12) 5. Diploma/certificate 6. Degree and above | | |  | |  |
| Q106 | | What is your husband educational status? | | 1. Unable to read and write 2. Able to read and write 3. Elementary school(grade 1-8) 4. High school(grade 9-12) 5. Diploma/certificate 6. Degree and above | | |  | |  |
| Q107 | | What is your occupational status? | | 1. Government employee 2. Private employee 3. NGO employee 4. Running personal business 5. House wife 6. Jobless 7. Others | | |  | |  |
| Q107-2 | | Others please specify | | ______________ | | |  | |  |
| Q108 | | What is your husband educational status? | | 1. Government employee 2. Private employee 3. NGO employee 4. Running personal business 5. Farmer 6. Jobless 7. Others | | |  | |  |
| Q108-2 | | Others please specify | | _______________ | | |  | |  |
| Q109 | | Where is your residence? | | 1. Rural 2. Urban | | |  | |  |
| Q110 | | What is your monthly income? | | ___________ Ethiopian | | |  | |  |
| SECTION TWO: EDINBURGH POSTNATAL SCALE  Gestational age = ___________Total score=_________ | | | | | | | | | |
| Q.NO. | | Questions | | Alternative responses | | | Skip | | Code |
| Q201 | | I have been able to laugh and see the funny side of things: | | 0-As much as I always could  1-Not quite as much now  2-Definitely not so much now  3-Not at all | | |  | |  |
| Q202 | | I have look forward with enjoyment to things: | | 0-As much as I ever did  1-Rather less than I used to  2-Definitely less than I used to  3-Hardly at all | | |  | |  |
| Q203 | | I have blamed myself unnecessarily when things went wrong: | | 3-Yes, most of the time  2-Yes, some of the time  1-Not very often  0-No, never | | |  | |  |
| Q204 | | I have been anxious or worried for no good reason: | | 0-No, not at all  1-Hardly ever  2-Yes, sometimes  3-Yes, very often | | |  | |  |
| Q205 | | I have felt scared or panicky for no very good reason: | | 3-Yes, quite a lot  2-Yes, sometimes  1-No, not much  0-No, not at all | | |  | |  |
| Q206 | | Things have been getting on top of me: | | 3-Yes, most of the time I haven’t been able to cope at all  2-Yes, sometimes I haven’t been coping as well as usual  1-No, most of the time I have coped quite well  0-No, I have been coping as well as ever | | |  | |  |
| Q207 | | I have been so unhappy that I have had difficulty sleeping: | | 3-Yes, most of the time  2-Yes, sometimes  1-Not very often  0-No, not at all | | |  | |  |
| Q208 | | I have felt sad or miserable: | | 3-Yes, most of the time  2-Yes, quite often  1-Not very often  0-No, not at all | | |  | |  |
| Q209 | | I have so unhappy that I have been crying | | 3-Yes, most of the time  2-Yes quite often  1-Only occasionally  0-No, never | | |  | |  |
| Q210 | | The thought of harming myself has occurred to me: | | 3-Yes, quite often  2-Sometimes  1-Hardly ever  0-Never | | |  | |  |
| PART THREE:Obstetric history | | | | | | | | | |
| Q.NO. | | Questions | | Alternative responses | | | Skip | | Code |
| Q301 | | Have you been pregnant before? | | 1. No 2. Yes | | | If no skip to Q304 | |  |
| Q301-2 | | If yes what was the number of your previous pregnancy? | | _______________ | | |  | |  |
| Q302 | | Do you have history of abortion? | | 1. No 2. Yes | | |  | |  |
| Q302-2 | | If yes what was the pattern of the previous abortion? | | 1. Spontaneous 2. Induced | | |  | |  |
| Q303 | | Do you have history of stillbirth | | 1. No 2. Yes | | |  | |  |
| Q304 | | Is your current pregnancy is planned? | | 1. No 2. Yes | | |  | |  |
| Q305 | | What is the number of weeks of current pregnancy? | | __________ | | |  | |  |
| Q306 | | At what gestational age did you start ANC follow-up? | | __________ in week | | |  | |  |
| PART FOUR: Psychosocial history | | | | | | | | | |
| Q.NO. | | Questions | | | Alternative responses | | Skip | | Code |
| Q401 | | What is the feeling of your husband about your pregnancy? | | | 1. Happy(Good 2. Not happy(Poor) | |  | |  |
| Q402 | | How do you explain the support of your husband/the baby father to the health of the fetus and continuation of pregnancy? | | | 1. Very good 2. Good 3. Not good | |  | |  |
| Q403 | | For the last twelve months, is their emotionally disturbing factor to you? | | | 1. No 2. Yes | | If no skip to Q405 | |  |
| Q403-2 | | If so please specify emotionally disturbing factors to you: | | | 1. Educational status 2. Monthly income 3. Your occupation 4. Marital status 5. Relation to your partner 6. Social support 7. Your health 8. Others | |  | |  |
| Q403-3 | | Others please specify | | | _________ | |  | |  |
| Q404 | | What are the main factors making you worry about most of the time in relation to this pregnancy? | | | 1. Educational status 2. Monthly income 3. Your occupation 4. Marital status 5. Relation to your partner 6. Social support 7. Your health 8. Others | |  | |  |
| Q404-2 | | Others please specify | | | _______________ | |  | |  |
| Q405 | | Do you think your psychosocial problems addressed at the antenatal clinic? | | | 1. No 2. Yes | |  | |  |
| Q406 | | Do you have enough information about the pregnancy and expected labour? | | | 1. No 2. Yes | |  | |  |
| PART FIVE : OSLO social support scale  Total score = -------------- | | | | | | | | | |
| Q.NO. | | Questions | | | Alternative responses | | Skip | | Code |
| Q501 | | How many people are so close to you that you can count on them if you have serious problems? | | | 1. None 2. 1 or 2 3. 3 to 5 4. 6 or more | |  | |  |
| Q502 | | How much concern do people show in what you are doing? | | | 1. A lot of concern and interest   4- Some concern and interest  3- Uncertain  2- Little concern and interest   1. No concern and interest | |  | |  |
| Q503 | | How easy can you get practical help from neighbors if you should need it? | | | 5- Very easy  4- Easy  3- Possible  2- Difficult  1- Very difficult | |  | |  |
| PART SIX: History of violence | | | | | | | | | |
| Q.NO, | | Questions | | | Alternative responses | | Skip | | Code |
| Q601 | | Do you have violence committed on you? | | | 1. No 2. Yes | |  | |  |
| Q602 | | In your lifetime, did your partner/husband intentionally push, hit/slap, kick or causes any physical harm with the intent to cause fear or injury? | | | 1. No 2. Yes | |  | |  |
| Q602-2 | | During current pregnancy, did your partner/husband intentionally push, hit/slap, kick or causes any physical harm with the intent to cause fear or injury? | | | 1. No 2. Yes | |  | |  |
| Q603 | | In your lifetime, did your partner/husband undermine your sense of self-worth and/or self-esteem? | | | 1. No 2. Yes | |  | |  |
| Q603-2 | | During current pregnancy, did your partner/husband undermine your sense of self-worth and/or self-esteem? | | | 1. No 2. Yes | |  | |  |
| Q604 | | In your lifetime, did your partner/husband coerce or attempt to coerce any sexual contact or behavior without your consent? | | | 1. No 2. Yes | |  | |  |
| Q604-2 | | During current pregnancy, did your partner/husband coerce or attempting to coerce any sexual contact or behavior without your consent? | | | 1. No 2. Yes | |  | |  |
| PART SEVEN: History of clinical factors | | | | | | | | | |
| Q.NO. | | Questions | | Alternative responses | | | Skip | | Code |
| Q701 | | Did you have lifetime experience of depressive episode (defined as ever-experienced sadness or loss of interest in normal activities almost daily for two weeks or more)? | | 1. No 2. Yes | | |  | |  |
| Q702 | | Is there any family history of depressive episode? | | 1. No 2. Yes | | |  | |  |
| Q703 | | Do you have any chronic illness? | | 1. No 2. Yes | | |  | |  |
| Q703-2 | | If yes, what chronic disease do you have? | | 1. Diabetes mellitus 2. Hypertension 3. HIV/AIDS 4. Others | | |  | |  |
| Q703-3 | | Others please specify | | ______________ | | |  | |  |
| PART EIGHT: History of substance use  INTRODUCTION  I am going to ask some questions about your experience of using these substances across your lifetime and in the past twelve months. These substances can be smoked, swallowed, sniffed, injected or taken in the form of pills. A doctor may prescribe some of the substances listed (like Amphetamines, sedatives, and pain medications). For this interview, we will not record medications that used as prescribed by your doctors. However, if you have taken such medicines for reasons other than prescription, or taken them more frequently or at higher doses than prescribed, please let me know. While we are also interested in knowing about your use of various illegal drugs, please be assured that information on such use will treated as strictly confidential. | | | | | | | | | |
| Q.NO. | Questions | | Alternative responses | | | Skip | | Code | |
| Q801 | In your lifetime, which of the following substances have you ever used?(NON-MEDICAL USE ONLY) | | 1. No, I don’t use 2. Tobacco products (cigarettes, chewing tobacco, cigars, etc) 3. Alcoholics beverages(beer, wine, etc) 4. Cannabis(Marijuana, pot, grass, hash, etc) 5. Cocaine(Coke, crack, etc) 6. Amphetamine type stimulants(chat) 7. Others | | |  | |  | |
| Q801-2 | If others, please specify | | __________________ | | |  | |  | |
| Q802 | During current pregnancy, which of the following substances have you ever used?(NON-MEDICAL USE ONLY) | | 1. No, I don’t use 2. Tobacco products (cigarettes, chewing tobacco, cigars, etc) 3. Alcoholics beverages(beer, wine, etc) 4. Cannabis(Marijuana, pot, grass, hash, etc) 5. Cocaine(Coke, crack, etc) 6. Amphetamine type stimulants(chat) 7. Others | | |  | |  | |
| Q802-2 | If others, please specify | | ______________ | | |  | |  | |

Name of data collector: ___________________________

Signature: ________________________

Date and time of collection: _________________

**Amharic version questionnaire**

**መመሪያ**፡መጠይቁ ሰባት ክፍል አለው፡፡ቃለመጠይቁን ለማጠናቀቅ 20 ደቂቃ የሚወስድ ሲሆን ሁሉንም ጥያቂዎች ለመመለስ ይሞክሩ፡፡ለትግስትዎእናመሰግናለን፡፡

| **ክፍልአንድ፡የተጠያቂውየግልመረጃየመለያቁጥር፡--------** | | | | | | | | |  |  |  |
| --- | --- | --- | --- | --- | --- | --- | --- | --- | --- | --- | --- |
| **ተ/ቁ** | | **ጥያቂዎች** | **መልስሊሆኑየሚችሉአማራጮች** | **እለፍ** | | **ኮድ** | | |  |  |  |
| ጥ101 | | እድሜዎስንትነው? | ____________ |  | |  | | |  |  |  |
| ጥ102 | | ሀይማኖትዎምንድንነው? | 1. ኦርቶዶክስ 2. ሙስሊም 3. ፕሮቴስታንት 4. ካቶሊክ 5. ሌሎች |  | |  | | |  |  |  |
| ጥ103 | | ብሄርዎምንድንነው? | 1. አማራ 2. ኦሮሞ 3. ትግሬ 4. ሌሎች |  | |  | | |  |  |  |
| ጥ104 | | የጋብቻሁኔታዎምንድንነው? | 1. ያላገባች 2. ያገባችናአብራየምትኖር 3. ያገባችናአብራየማትኖር 4. የተፋታች 5. ባሏየሞተባት |  | |  | | |  |  |  |
| ጥ105 | | የት/ትደረጃዎምንድንነው? | 1. መፃፍናማንበብየማትችል 2. መፃፍናማንበብየምትችል 3. የመጀሪያደረጃት/ት(ከ 1-8ኛክፍል) 4. የሁለተኛደረጃት/ት(ከ 9-12ኛክፍል) 5. ዲፕሎማ/ሠርተፍኬት 6. ድግሪናከዚያበላይ |  | |  | | |  |  |  |
| ጥ106 | | የባለቤትዎየትምህርትደረጃምንድንነው? | 1. መፃፍናማንበብየማይችል 2. መፃፍናማንበብየማይችል 3. የመጀሪያደረጃት/ት(ከ 1-8ኛክፍል) 4. የሁለተኛደረጃት/ት(ከ 9-12ኛክፍል) 5. ዲፕሎማ/ሠርተፍኬት 6. ድግሪናከዚያበላይ |  | |  | | |  |  |  |
| ጥ107 | | የስራዎትአይነትምንድንነው? | 1. የመንግስትተቀጣሪ 2. መንግስታዊያልሆነድርጅትተቀጣሪ 3. የግልድርጅትተቀጣሪ 4. የንግድስራ 5. የቤትእመቤት 6. ሥራየሌለው 7. ሌሎች |  | |  | | |  |  |  |
| ጥ107-2 | | ሌሎችንከመረጡይግለጡ? | --------------------------- |  | |  | | |  |  |  |
| ጥ108 | | የባለቤትዎየስራአይነትምንድንነው? | 1. የመንግስትተቀጣሪ 2. መንግስታዊያልሆነድርጅትተቀጣሪ 3. የግልድርጅትተቀጣሪ 4. የንግድስራ 5. የግብርናሥራ 6. ሥራየሌለው 7. ሌሎች |  | |  | | |  |  |  |
| ጥ109 | መኖሪያዎየትነው? | 1. ገፀር 2. ከተማ |  | |  | | |  |  |  |  |
| ጥ110 | የወርገቢዎምንያህልነው? | --------------------------- የኢትዮጵያብር |  | |  | | |  |  |  |  |
| **ክፍልሁለት፡የእርግዝናእናየወሊድጉዳዮችመረጃ** | | | | | | | |  |  |  |  |
| **ተ/ቁ** | **ጥያቄዎች** | **መልስሊሆኑየሚችሉአማራጮች** | **እለፍ** | | **ኮድ** | | |  |  |  |  |
| ጥ201 | ከዚህበፊትአርግዘውያውቃሉ? | 1. የለም 2. አዎን | የለምካሉወደጥ204 ይለፉ | |  | | |  |  |  |  |
| ጥ201-2 | አዎንካሉስንትጊዜአርግዘውያውቃሉ? | ---------------------- |  | |  | | |  |  |  |  |
| ጥ202 | ከዚህበፊትውርጃኖሮዎትያውቃል? | 1. የለም 2. አዎን |  | |  | | |  |  |  |  |
| ጥ202-2 | አዎንካሉየነበረውየውርጃሁኔታምንይመስልነበር? | 1. ባልታወቀምክንያትያጋጠመ 2. ታስቦበትየተደረገ |  | |  | | |  |  |  |  |
| ጥ203 | ከዚህበፊትበነበረዎእርግዝናሞቶየተወለደህፃንአጋጥሞዎትያውቃል? | 1. የለም 2. አዎን |  | |  | | |  |  |  |  |
| ጥ204 | ያሁኑንእርግዝናአቅደውበታል/ይፈልጉታል? | 1. የለም 2. አዎን |  | |  | | |  |  |  |  |
| ጥ205 | ያሁኑእርግዝናዎስንትሳምንትሆኖታል? | ---------------ሳመንት |  | |  | | |  |  |  |  |
| ጥ206 | በስንተኛየእርግዝናጊዜዎነውየነፍሰጡርክትትልየጀመሩት ? | ---------------- ሳምንት |  | |  | | |  |  |  |  |
| **ክፍል ሦስት ፡ የኢደንበርግ ፖስትናታል ዲፕረሽን ስኬል የአማረኛ ትርጉም**  **የእርግዝናዎ እድሜ በሳምንት፡ ----------------- ጠቅላላ ድምር፡---------** | | | | | | | |  |  |  |  |
| **ተ/ቁ** | **ጥያቂዎች** | **መልስ ሊሆኑ የሚችሉ አማራጮች** | **እለፍ** | | **ኮድ** | | |  |  |  |  |
| ጥ301 | ባለፈው ሳምንት ዉስጥ የነገሮችን አስቂኝ ሁኔታ በማየት ለመሳቅ ችለው ነበር? | 1. ሁልግዜ የማደርገውን ያህል 2. ባሁኑ ግዜ የድሮውን ያህል አልችልም 3. በርግጠኝነት የድሮውን ያህል አልችልም ነበር 4. ፍፁም አልችልም ነበር |  | |  | | |  |  |  |  |
| ጥ302 | ባለፈው ሳምንት ውስጥ ነገሮችን በደስታ/በናፍቆት/ ሲጠባብቁ ነበር? | 1. ከዚህ በፊት እንደማደርገው ያህል 2. በመጠኑ በፊት ከማደርገው በቀነሰ ሁኔታ 3. በርግጠኝነት በፊት ከማደርገው ባነሰ ሁኔታ 4. በፍፁም አልጠባበቅም ነበር |  | |  | | |  |  |  |  |
| ጥ303 | ባልፈው ሳምንት ዉስጥ አንዳንድ ነገሮች ሳይሳኩ ሲቀር ያለአግባብ እራስዎን ይወቅሱ ነበር? | 1. አዎን አብዛኛውን ጊዜ 2. አዎን አንዳንድ ጊዜ 3. በጣም ጥቂት ጊዜ 4. አይ በፍጹም አልነበረም |  | |  | | |  |  |  |  |
| ጥ304 | ባለፍው ሳምንት ዉስጥ ያለበቂ ምክንያት ሲጨነቁ/ሲሰጉ ነበር? | 1. አዎን አብዛኛውን ጊዜ 2. አዎን አንዳንድ ጊዜ 3. እምብዛም አያጋጥመኝም 4. አይ አጋጥሞኝ አያውቅም |  | |  | | |  |  |  |  |
| ጥ305 | ባለፈው ሳምንት ዉስጥ ያለበቂ ምክንያት የመፍራት፤ የመሸበር፤ ድንግጥ-ድንግጥ የማለት ስሜት ይሰማዎት ነበር? | 1. አዎን ብዙውን ጊዜ 2. አዎን አንዳንድ ጊዜ 3. በጣም ጥቂት ጊዜ 4. አይ ፍጡም አልነበረም |  | |  | | |  |  |  |  |
| ጥ306 | ባለፈው ሳምንት ዉስጥ ነገሮች ሁሉ ከአቅም በላይ እየሆኑብዎት ነበር? | 1. አዎ ብዙ ጊዜ መቋቋም አልቻልኩም 2. አዎ አንዳንድ ጊዜ መቋቋም አልቻልኩም 3. የለም ብዙ ጊዜ መቋቋም ችያለሁ 4. የለም እንደወትሮየ መቋቋም ችያለሁ |  | |  | | |  |  |  |  |
| ጥ307 | ባለፈው ሳምንት ውስጥ ደስታ በጣም ከማጣት የትነሳ የእንቅልፍ ችግር ነበረብዎት? | 1. አዎን አብዛኛውን ጊዜ 2. አዎን አንዳንድ ጊዜ 3. የለም በጣም ጥቂት ጊዜ 4. የለም ፈፅሞ አልነበረም |  | |  | | |  |  |  |  |
| ጥ308 | ባለፈው ሳምንት ውስጥ የመከፋት ወይም የመረረ ሀዘን ይሰማዎት ነበር? | 1. አዎን አብዛኛውን ጊዜ 2. አዎን በመጠኑ ብዙ ጊዜ 3. የለም በጣም ብዙ ጊዜ አልነበረም 4. የለም ፈፅሞ አልነበረም |  | |  | | |  |  |  |  |
| ጥ309 | ባለፈው ሳምንት ውስጥ ደስታ በጣም ከማጣት የተነሳ አልቅሰው ነበር? | 1. አዎን አብዛኛውን ጊዜ 2. አዎን በመጠኑ ብዙ ጊዜ 3. አልፎ -አልፎ ብቻ 4. የለም ፈፅሞ አልነበረም |  | |  | | |  |  |  |  |
| ጥ310 | ባለፈው ሳምንት ውስጥ በህይዎትዎ ላይ ጉዳት ለማድርስ አስበው ነበር? | 1. አዎን ብዙ ጊዜ 2. አንዳንድ ጊዜ 3. እምብዛም አስቤ አላውቅም 4. በፍጹም አላሰብኩም |  | |  | | |  |  |  |  |
| **ክፍል አራት፡ የስነ-ልቦናዊና ማህበራዊ ጉዳዮች መረጃ** | | | | | | | | |  |  |  |
| **ተ/ቁ** | | **ጥያቄዎች** | **መልስ ሊሆኑ የሚችሉ አማራጮች** | **እለፍ** | | **ኮድ** | | |  |  |  |
| ጥ401 | | እርጉዝ በመሆንዎ የባለቤትዎ ስሜት ምንድን ነው? | 1. ደስተኛ(ጥሩ) 2. ደስተኛ አይደለም(ጥሩ ያልሆነ) |  | |  | | |  |  |  |
| ጥ402 | | ባለቤትዎ(የልጁ አባት) ለጽንሱ ጤንነትና እርግዝናው እንዲቀጥል እያደረጉ ያለውን ጥረት እንዴት ይገልጡታል? | 1. በጣም ጥሩ ነው 2. ጥሩ ነው 3. ጥሩ አይደለም |  | |  | | |  |  |  |
| ጥ403 | | ባለፉት አስራ ሁለት ወራት ውስት ስሜትዎን ወይም አእምሮዎን የሚረብሹ ነገሮች አሉ? | 1. የለም 2. አዎን | የለም ካሉ ወደ ጥ405 ይለፉ | |  | | |  |  |  |
| ጥ403-2 | | መልስዎ አዎን ከሆነ አእምሮዎን የሚረብሹ ጉዳዮችን ምንድን ናቸው? | 1. የትምህርት ደረጃዎ 2. የወር ገቢዎ 3. የስራዎ አይነት 4. የትዳር ሁኔታዎ 5. ከባለቤትዎ ጋር ያለዎት ግንኙነት 6. የሕብረተሰቡ ድጋፍ 7. የጤናዎ ጉዳይ 8. ሌሎች |  | |  | | |  |  |  |
| ጥ403-3 | | ሌሎችን ከመረጡ እባክዎን ይግለጹ? | ------------------------- |  | |  | | |  |  |  |
| ጥ404 | | ካሁኑ እርግዝናዎ ጋር በተየያዘ በዋነኝነት የሚያስጨንቆዎት ጉዳዮች ምንድን ናቸው? | 1. የትምህርት ደረጃዎ 2. የወር ገቢዎ 3. የስራዎ አይነት 4. የትዳር ሁኔታዎ 5. ከባለቤትዎ ጋር ያለዎት ግንኙነት 6. የሕብረተሰቡ ድጋፍ 7. የጤናዎ ጉዳይ 8. ሌሎች | |  | |  | | |  |  |
| ጥ405 | | የእርግዝና ምርመራ ክትትል በማድረግዎ ለስነ-ልቦናዊና እና ማህበራዊ ችግሮች ያገኙት መፍትሄ አለ ብለው ያስባሉ? | 1. የለም 2. አዎ |  | | | |  | | | |
| ጥ406 | | ስለ እርግዝናዎና ውደፊት ስለሚጠበቀው የምጥ ሁኔታዎች በቂ መረጃ አግኝተዋል? | 1. የለም 2. አዎ |  | | | |  | | | |
| **ክፍል አምስት፡ ኦስሎ የማኅበረሰብ ድጋፍ መለኪያ አማረኛ ትርጉም**  **ጠቅላላ ድምር ---------------** | | | | | | | |  |  |  |  |
| **ተ/ቁ** | | **ጥያቄዎች** | **መልስ ሊሆኑ የሚችሉ አማራጮች** | **እለፍ** | | | | **ኮድ** | | | |
| ጥ501 | | በችግር ጊዜ ይደርሱልኛል ብለው የሚተማመኑባቸው ስንት ሰዎች አሉ? | 1. ምንም 2. 1 ወይም 2 3. ከ 3-5 4. 6 ና ከዚያ በላይ |  | | | |  | | | |
| ጥ502 | | እርስዎ ለሚሰሯቸው ስራዎች ማህበረሰቡ ምን ያህል ይጨነቃል? | 1. ከፍተኛ ጭንቀትና ፍላጎት 2. መጠነኛ ጭንቀትና ፍላጎት 3. እርግጠኛ አይደለሁም 4. ዝቅተኛ ጭንቀትና ፍላጎት 5. ምንም አይጨነቁም |  | | | |  | | | |
| ጥ503 | | በአቅራቢያዎ/ከጎረቤትዎ ከሚገኙ ህብረተሰቦች ተግባራዊ ድጋፍ ለማግኘት ምን ያህል ቀላል ነው ብለው ያስባሉ? | 1. በጣም ቀላል 2. ቀላል 3. መካከለኛ 4. ከባድ 5. በጣም ከባድ |  | | | |  | | | |
| **ክፍል ስድስት፡ ስለ ጭቆና መረጃ** | | | | | | | |  |  |  |  |
| **ተ/ቁ** | | **ጥያቄዎች** | **መልስ ሊሆኑ የሚችሉ አማራጮች** | **እለፍ** | | | | **ኮድ** | | | |
| ጥ601 | | በህይዎት ዘመንዎ በእርስዎ ላይ ጭቆና ደርሶብዎት ያውቃል? | 1. የለም 2. አዎን | የለም ካሉ ወደ ጥ701 ይለፉ | | | |  | | | |
| ጥ602 | | በህይዎት ዘመንዎ ባለቤትዎ/የትዳር አጋርዎ በእርስዎ ላይ ለማስፈራራት ወይም ጉዳት ለማድረስ ብሎ ገፍቶዎት፣በጥፊ/በቡጢ መቶዎት፣ ረግጦዎት ወይም በማንኛውም መልኩ ጉዳት አድርሶብዎት ያውቃል? | 1. የለም 2. አዎን | የለም ካሉ ወደ ጥ603 ይለፉ | | | |  | | | |
| ጥ602-2 | | አዎን ካሉ ባሁኑ የእርግዝና ጊዜዎ ባለቤትዎ/የትዳር አጋርዎ በእርስዎ ላይ ለማስፈራራት ወይም ጉዳት ለማድረስ ብሎ ገፍቶዎት፣በጥፊ/በቡጢ መቶዎት፣ ረግጦዎት ወይም በማንኛውም መልኩ ጉዳት አድርሶብዎት ያውቃል? | 1. የለም 2. አዎን |  | | | |  | | | |
| ጥ603 | | በህይዎት ዘመንዎ ባለቤትዎ/የትዳር አጋርዎ ያለዎትን በራስ መተማመንና ጥንካሬ አመንምኖት ያውቃል ? | 1. የለም 2. አዎን | የለም ካሉ ወደ ጥ604 ይለፉ | | | |  | | | |
| ጥ603-2 | | አዎን ካሉ ባሁኑ የእርግዝና ጊዜዎ ባለቤትዎ/የትዳር አጋርዎ ያለዎትን በራስ መተማመንና ጥንካሬ አመንምኖት ያውቃል? | 1. የለም 2. አዎን |  | | | |  | | | |
| ጥ604 | በህይዎት ዘመንዎ ባለቤትዎ/የትዳር አጋርዎ ያለርስዎ ፍቃድ ፆታዊ ባህሪ ወይም ንክኪ አስገድዶ አድርጎ ወይም ለማድረግ ሞክሮ ያውቃል? | 1. የለም 2. አዎን | የለም ካሉ ወደ ጥ701 ይለፉ | | | |  | | | |  |
| ጥ604-2 | አዎን ካሉ ባሁኑ የእርግዝና ጊዜዎ ባለቤትዎ/የትዳር አጋርዎ ያለርስዎ ፍቃድ ፆታዊ ባህሪ ወይም ንክኪ አስገድዶ አድርጎ ወይም ለማድረግ ሞክሮ ያውቃል? | 1. የለም 2. አዎን |  | | | |  | | | |  |
| ጥ601-3 | | ሌሎች ከመረጡ እባክዎን ይግለጹ? | ----------------------- |  | | | |  | | | |
| **ክፍል ሰባት፡ ስለ ክሊኒካል ችግሮች መረጃ** | | | | | | | |  |  |  |  |
| **ተ/ቁ** | | **ጥያቄዎች** | **መልስ ሊሆኑ የሚችሉ አማራጮች** | **እለፍ** | | | | **ኮድ** | | | |
| ጥ701 | | በህይወትዎ ውስጥ የድብርት ህመም/ ማለትም ሁለትና ከዚያ በላይ ለሚሆኑ ሳምንታት በቀን ውስት አብዛኛውን ጊዜ የማዘን ስሜት ወይም እለት ከእለት በሚያደርጓአቸው ስራዎች ላይ ፍላጎት ማጣት/ ተከስትዎበት ያውቃል? | 1. የለም 2. አዎን |  | | | |  | | | |
| ጥ702 | | በቤተሰብዎት ውስጥ የድብርት በሽታ ተከስቶ ያውቃል? | 1. የለም 2. አዎን |  | | | |  | | | |
| ጥ703 | | በሐኪም የተነገሮት ክትትል የሚያስፈልገው ስር የሰደደ የጤና መታወክ /ለምሳሌ የደም ግፊት ፣ የስኩር በሽታ/ አለብዎት? | 1. የለም 2. አዎን |  | | | |  | | | |
| ጥ703-2 | | አዎን ካሉ እባኮትን ይጥቀሱ(ከሕክምና ካርዳቸው ይሞላ) | -------------------------- |  | | | |  | | | |
| **ክፍል ስምንት፡ ስለ እፅ ተጠቃሚዎች መረጃ**  **መግቢያ፡** በህይዎት ጊዜዎና ባለፉት አስራ ሁለት ወራት ስለነዚህ እጾች መጠቀም ጥያቄዎችን ልንጠይቀዎት ነው፡፡ እነዚህ ነገሮች የሚጨሱ ፣ የሚዋጡ ፣ የሚሸተቱ ፣ መርፌ ሚወጉ ወይም በክኒን መልኩ የሚወሰዱ ሊሆኑ ይችላሉ፡፡ አንዳንድ መድኃኒቶች በዶክተር የታዘዙ ሊሆኑ ይችላሉ/ ለምሳሌ አንፊታሚን ፣ የእንቅልፍ ክኒን ፣ የህመም ማስታገሻ መድኃኒቶች/ ለዚህ ቃለ መጠይቅ በዶክተር የታዘዙ መድኃኒቶች ግምት ውስጥ አናስገባም፡፡ ሆኖም ግን እነዚህ መድኃኒቶች ከታዘዘልዎት አላማ በተጨማሪ በከፍተኛ መጠን በየጊዜው የሚወሰዱ ከሆነ ያሳውቁኝ፡፡ አንዳንድ ህገወጥ የሆነ የመድኃኒት አጠቃቀምዎን ያለስጋት ይግለጹ፡፡ ይህም መረጃ በፍጹም ሚስጢራዊ እንደሚሆን ይወቁ፡፡ | | | | | | | |  |  |  |  |
| **ተ/ቁ** | | **ጥያቄዎች** | **መልስ ሊሆኑ የሚችሉ አማራጮች** | **እለፍ** | | | | **ኮድ** | | |  |
| ጥ801 | | በህይዎትዎ ውስጥ ከዚህ በታች ከተዘረዘሩት እፅች ውስጥ የትኞቹን ተጠቅመዋል? /በሕክምና ያልታዘዙትን ብቻ/ | 1. የለም አልተጠቀምኩም 2. የትምባሆ ውጤቶች/ሲጋራ፣የሚታኘክ፣ሱረት ወዘተ/ 3. የአልኮል መጠጦች/ቢራ፣ ወይን ወዘተ 4. ካናቢስ/ማሪዋና ፣ ፓች ፣ ግራስ ፣ ሀሽሽ ወዘተ 5. ኮኬይን/ኮክ ፣ ክራክ ወዘተ/ 6. የአምፊታሚን አይነት አነቃቂ እፆች/ጫት/ 7. ሌሎች |  | | | |  | | |  |
| ጥ801-2 | | ሌሎችን ከመረጡ እባክዎት ይግለለጹ? |  |  | | | |  | | |  |
| ጥ802 | | ባሁኑ ዝርግዝናዎ ከዚህ በታች ከተዘረዘሩት እጾች ውስጥ የትኞቹን ተጠቅመዋል ?/በሕክምና ያልታዘዙትን ብቻ/ | 1. የለም አልተጠቀምኩም 2. የትምባሆ ውጤቶች/ሲጋራ ፣ የሚታኘክ ፣ ሱረተ ወዘተ/ 3. የአልኮል መጠጦች/ቢራ ፣ ወይን ወዘተ/ 4. ካናቢስ/ማሪዋና ፣ ፓች ፣ ግራስ ፣ ሀሽሽ ወዘተ/ 5. ኮኬይን/ኮክ ፣ ክራክ ወዘተ/ 6. የአምፊታሚን አይነት አነቃቂ እፆች/ጫት/ 7. ሌሎች |  | | | |  | | |  |
| ጥ802-2 | | ሌሎችን ከመረጡ እባክዎን ይግለጡ? |  |  | | | |  | | |  |

**ስለትእግስትዎ በድጋሚ እናመሰግናለን፡፡**

**የመረጃ ሰብሳቢው ስም፡ -------------------------- ፊርማ፡ ---------------**

**ቃለ መጠይቁ የተደረገበት ቀንና ሰአት፡ -------------------------**
