# Supplementary material for: Prevalence of depression and associated factors among pregnant women attending antenatal care in public health institutions of Awabale Woreda, East Gojjam Zone, Northwestern Ethiopia: A cross-sectional study
Source: PLoS One. 2022 Oct 13;17(10):e0271876. doi: 10.1371/journal.pone.0271876 (PMC9560138; doi:10.1371/journal.pone.0271876)
Supplement: Attachment — Submitted filename: PONE-D-21-01660_R3.pdf [file pone.0271876.s008.pdf]

PLOS ONE

Prevalence of Depression and Associated Factors among Pregnant Women Attending Antenatal Care in Public Health Institutions of Awabale Woreda, East Gojjam Zone, Northwestern Ethiopia: a cross-sectional study

--Manuscript Draft--

**Manuscript Number:**	PONE-D-21-01660R3
**Article Type:**	Research Article
**Full Title:**	Prevalence of Depression and Associated Factors among Pregnant Women Attending Antenatal Care in Public Health Institutions of Awabale Woreda, East Gojjam Zone, Northwestern Ethiopia: a cross-sectional study
**Short Title:**	Prevalence of Depression and Associated Factors among Pregnant Women Attending Antenatal Care
**Corresponding Author:**	Bewket Yeserah Aynalem<br>Debremarkos University<br>Debremarkos, ETHIOPIA
**Keywords:**	Depression; antenatal depression; antenatal care; Awabel Woreda
**Abstract:**	Background: Antenatal depression is a serious health problem and has negative consequences for the mother, fetus, and the entire family. However, it is a neglected component of care for women in pregnancy. The purpose of this study was to assess the prevalence of depression and associated factors among pregnant women attending antenatal clinics in public health institutions, in the Awabale Woreda<br><br>Method: An institutional-based cross-sectional study was conducted in 2018 and a stratified sampling technique was used to select the study health institutions. A structured questionnaire was used to collect data. Edinburgh Postnatal Depression Scale was used to declare the presence of antenatal depression with a cut point score of 13 and above.<br><br>Result: This study showed that 63(17.8%) pregnant mothers had antenatal depression. Women who were running their business were 85% reduced to develop antenatal depression than housewives [AOR=0.15(0.001-0.25)]. Pregnant women had high school and above educational level 18 times higher odds of developing antenatal depression than women who had no formal education [AOR18.15 (2.73-120.76)]. Women who had poor husband feeling on the current pregnancy were 4.94 more likely to develop antenatal depression than women who had good partner feeling on the current pregnancy [AOR=4.94(95%CI: 1.78-13.72)]. Women who had a history of depression were 8.2 times to develop antenatal depression than women who had no history of depression [AOR=8.22 (95%CI: 2.87-23.57)].<br>Conclusion: This study revealed that one-fifth of pregnant women developed antenatal depression. Women's occupational status, educational status, previous history of depression, and poor husband feeling on the current pregnancy were the significant factors of antenatal depression.
**Order of Authors:**	Alemayehu Bantie
	getachew mullu kassa
	Haymanot Zeleke
	Liknaw Bewket Zeleke
	Bewket Yeserah Aynalem
**Response to Reviewers:**	thank you for your constructive comments and suggestions. you teach me a lot
**Additional Information:**	
**Question**	**Response**
**Financial Disclosure**	Debre Markos University was the funding agency for data collection

Powered by Editorial Manager® and ProduXion Manager® from Aries Systems Corporation

Enter a financial disclosure statement that describes the sources of funding for the work included in this submission. Review the submission guidelines for detailed requirements. View published research articles from *PLOS ONE* for specific examples.<br><br>This statement is required for submission and **will appear in the published article** if the submission is accepted. Please make sure it is accurate.<br><br>**Unfunded studies**<br>Enter: *The author(s) received no specific funding for this work.*<br><br>**Funded studies**<br>Enter a statement with the following details:<br>• Initials of the authors who received each award<br>• Grant numbers awarded to each author<br>• The full name of each funder<br>• URL of each funder website<br>• Did the sponsors or funders play any role in the study design, data collection and analysis, decision to publish, or preparation of the manuscript?<br>• **NO** - Include this sentence at the end of your statement: *The funders had no role in study design, data collection and analysis, decision to publish, or preparation of the manuscript.*<br>• **YES** - Specify the role(s) played.<br><br>* typeset	
**Competing Interests**<br><br>Use the instructions below to enter a competing interest statement for this submission. On behalf of all authors, disclose any competing interests that could be perceived to bias this work—acknowledging all financial support and any other relevant financial or non-financial competing interests.<br><br>This statement is required for submission and **will appear in the published article** if	The authors declare that they have no competing interests.

the submission is accepted. Please make sure it is accurate and that any funding sources listed in your Funding Information later in the submission form are also declared in your Financial Disclosure statement.<br><br>View published research articles from *PLOS ONE* for specific examples.<br><br>**NO authors have competing interests**<br><br>Enter: *The authors have declared that no competing interests exist.*<br><br>**Authors with competing interests**<br><br>Enter competing interest details beginning with this statement:<br><br>*I have read the journal's policy and the authors of this manuscript have the following competing interests: [insert competing interests here]*<br><br>* typeset	
**Ethics Statement**<br><br>Enter an ethics statement for this submission. This statement is required if the study involved:<br><br>• Human participants<br>• Human specimens or tissue<br>• Vertebrate animals or cephalopods<br>• Vertebrate embryos or tissues<br>• Field research<br><br>Write "N/A" if the submission does not require an ethics statement.<br><br>General guidance is provided below. Consult the submission guidelines for detailed instructions. **Make sure that all information entered here is included in the Methods section of the manuscript.**	The study was approved by the Institutional Health Research Ethics Review Committee of Debre Markos University, College of health science. An official letter was written from Debre Markos University to the selected health institutions. The participants enrolled in the study were informed about the study objectives, expected outcomes, benefits, and the risks associated with it. Signed written consent was taken from the participants before the interview.

Powered by Editorial Manager® and ProduXion Manager® from Aries Systems Corporation

**Format for specific study types**<br><br>**Human Subject Research (involving human participants and/or tissue)**<br>• Give the name of the institutional review board or ethics committee that approved the study<br>• Include the approval number and/or a statement indicating approval of this research<br>• Indicate the form of consent obtained (written/oral) or the reason that consent was not obtained (e.g. the data were analyzed anonymously)<br><br>**Animal Research (involving vertebrate animals, embryos or tissues)**<br>• Provide the name of the Institutional Animal Care and Use Committee (IACUC) or other relevant ethics board that reviewed the study protocol, and indicate whether they approved this research or granted a formal waiver of ethical approval<br>• Include an approval number if one was obtained<br>• If the study involved *non-human primates*, add *additional details* about animal welfare and steps taken to ameliorate suffering<br>• If anesthesia, euthanasia, or any kind of animal sacrifice is part of the study, include briefly which substances and/or methods were applied<br><br>**Field Research**<br><br>Include the following details if this study involves the collection of plant, animal, or other materials from a natural setting:<br>• Field permit number<br>• Name of the institution or relevant body that granted permission	
**Data Availability**<br><br>Authors are required to make all data underlying the findings described fully available, without restriction, and from the time of publication. PLOS allows rare exceptions to address legal and ethical concerns. See the PLOS Data Policy and FAQ for detailed information.	No - some restrictions will apply

A Data Availability Statement describing where the data can be found is required at submission. Your answers to this question constitute the Data Availability Statement and **will be published in the article**, if accepted.<br><br>**Important:** Stating 'data available on request from the author' is not sufficient. If your data are only available upon request, select 'No' for the first question and explain your exceptional situation in the text box.<br><br>Do the authors confirm that all data underlying the findings described in their manuscript are fully available without restriction?	
**Describe where the data may be found in full sentences. If you are copying our sample text, replace any instances of XXX with the appropriate details.**<br><br>• If the data are **held or will be held in a public repository**, include URLs, accession numbers or DOIs. If this information will only be available after acceptance, indicate this by ticking the box below. For example: *All XXX files are available from the XXX database (accession number(s) XXX, XXX.).*<br>• If the data are all contained **within the manuscript and/or Supporting Information files**, enter the following: *All relevant data are within the manuscript and its Supporting Information files.*<br>• If neither of these applies but you are able to provide **details of access elsewhere**, with or without limitations, please do so. For example:<br><br>*Data cannot be shared publicly because of [XXX]. Data are available from the XXX Institutional Data Access / Ethics Committee (contact via XXX) for researchers who meet the criteria for access to confidential data.*<br><br>*The data underlying the results presented in the study are available from (include the name of the third party*	All relevant data are within the manuscript.

and contact information or URL).<br>• This text is appropriate if the data are owned by a third party and authors do not have permission to share the data.<br><br>* typeset	
Additional data availability information:	

Powered by Editorial Manager® and ProduXion Manager® from Aries Systems Corporation

Manuscript Click here to access/download;Manuscript;manuscript.docx

Prevalence of Depression and Associated Factors among Pregnant Women Attending Antenatal Care in Public Health Institutions of Awabale Woreda, East Gojjam Zone, Northwestern Ethiopia: a cross-sectional study

Alemayehu Bantie[1], Getachew Mulu kassa[2], Haymanot Zeleke[2], Liknaw Bewket Zeleke[2], Bewket Yeserah Aynalem[2]*

[1] Debre Markos, Ethiopia

[2] Health Science College, Debre Markos University, Debre Markos, Ethiopia

*Corresponding author:

Email: by123bewket@gmail.com

Abstract

Background: Antenatal depression is a serious health problem and has negative consequences for the mother, fetus, and the entire family. However, it is a neglected component of care especially bay health care providers for women in pregnancy. The purpose of this study was to assess the prevalence of depression and associated factors among pregnant women attending antenatal clinics in public health institutions, in the Awabale Woreda

Method: An institutional-based cross-sectional study was conducted in 2018 and a stratified sampling technique was used to select the study health institutions. Stratified sampling was undertaken to select the study of health institutions. All seven public health institutions in Awabale District were included to select 393 mothers and the sample size was proportionally allocated based on the number of target mothers. We used EpiData version 3.1software for data entry and SPSS version 20 software for cleaning and analysis. Bivariable and multivariable logistic regressions analyses were done to identify factors associated with Antenatal depression. V[illegible]ables with a p-value less than 0.05 were taken as significant variables. Edinburgh Postnatal Depression

Scale was used to declare the presence of antenatal depression with a cut point score of 13 and above.

Result: This study showed that 63(17.8%) pregnant mothers had antenatal depression. Women who were employees were 85% reduced to develop antenatal depression than housewives [AOR=0.15(0.001-0.25)]. Pregnant women had high school and above educational level 18 times higher odds of developing antenatal depression than women who had no formal education [AOR18.15 (2.73-120.76)].
Women who had poor husband feeling on the current pregnancy were 4.94 more likely to develop antenatal depression than women who had good partner feeling on the current pregnancy [AOR=4.94(95%CI: 1.78-13.72)]. Women who had a history of depression were 8.2 times to develop antenatal depression than women who had no history of depression [AOR=8.22 (95%CI: 2.87-23.57)].
Conclusion: This study revealed that approximately one-fifth of pregnant women developed antenatal depression. Women's occupational status, educational status, previous history of depression, and poor husband feeling on the current pregnancy were the significant factors of antenatal depression.

Keywords: Depression; Antenatal depression; Antenatal care; Awabel Woreda

Introduction

Pregnancy is the period from the fertilization of the egg by sperm to the delivery of the fetus and usually lasts 40 weeks. Starting from the last normal menstrual period, it is divided into three trimesters, each lasting three months[1]. It is a distinctive social and biological event in a woman's life [2, 3].

A depressive disorder is an illness that involves the body, mood, and thoughts in which the person has unrelenting sentiments of unhappiness and irrelevance and a lack of aspiration to engage in previously enjoyable activities that last more than two weeks. When these sentiments last for a short period, it may be a case of feeling sadness[4]. It is the most frequent expressive disorder in women and the general population, and one in every five people has depression with more than the twofold increased incidence in

women than men [2]. Psychiatric history, stressful life events, lack of social support, teenage pregnancy, low educational level, low income, and violence against women are the factors that make depression prevalent in pregnant women [3, 5, 6].

Antenatal depression means depression that starts from conception to delivery[7]. Pregnant women experience symptoms similar to general depression and may interfere with their normal day-to-day activities. It may occur at any stage of pregnancy and can be a reaction to the pregnancy itself, due to health issues, major life stresses, genetic and biochemical basis, or due to a continuation or relapse of a pre-pregnancy condition[8]. Antenatal depression ends with poor fetal, infant development, and maternal outcomes like small for gestational age, prematurity, intrauterine growth problem; postnatal depression preeclampsia, anemia; educational problem, malnutrition, respiratory disorders, and mental retardation [9-13].

The majority of studies on the prevalence of antenatal depression and associated factors have been conducted in developed countries [14]. There is some studies, which examined the prevalence and associated factors of antenatal depression in low-income countries like Ethiopia; but the result of the studies were not consistent [15, 16] which showed farther study was needed. There are studies undertaken on antenatal depression [3]. Those studies have a limitation on addressing pregnant women in all age groups. In addition, they are likely to have a bias because most women with hindrance in their pregnancies are likely to seek antenatal care services in hospitals. There are studies, which were excluded illiterate women from the study[17].

Even though the Federal Democratic Republic of Ethiopia's National Mental Health Strategy promotes a decentralized approach in which mental health services are to be offered from local health institutions up to tertiary hospitals, the service is not being given as expected [18, 19]. Thus the purpose of this study was to assess the prevalence of depression and associated factors among pregnant women attending antenatal clinics in public health institutions, in Awabale Woreda.

Methods

Study area and period

This study was done in Awabale woreda, Amhara region, Northwest Ethiopia in 2018. Awabale woreda is the capital city of East Gojjam Zone in Amhara regional state, Northwest Ethiopia, which is located at 300 km from Addis Ababa, the capital city of Ethiopia, and 265 km from Bihar Dar, the capital city of Amhara region. Awabale woreda has a 146260 total population; about 37413 are females aged 15-49 years [20]. There are seven public health institutions eligible for this study in Awabale Woreda and Antenatal care is provided in all public health institutions. The Woreda has an annual ANC plan of 5590(1309 in Lumame health center, 648 in Shebela health center, 641 in Wejele health center, 665 in Lega health center, 901 in Yesenbet health center, 765 in Tsid-Maryam health center and 661 in Lumame primary hospital)[20].

Study design

An institutional-based cross-sectional study design was employed.

Study participants

All pregnant women attending antenatal care in Awabale Woreda public health institutions were the source population. All pregnant women attending antenatal care in selected public health institutions during the study period were the study population. Pregnant women at any age of gestation who come to the selected health institutions for antenatal care visits during the study period were included in the study and we excluded those who were critically ill during the data collection period.

Sample size

The required sample size was calculated using a single population proportion formula with the assumption of 31.2 % of depression during pregnancy from a study conducted in Adama, Ethiopia [16] and 5% marginal error (w), a standard Z score of 1.96 corresponding to a 95% confidence interval ($Z_{a/2}$),and 10% non-response rate.

$$n = \frac{(Za/_2)^2\, p\, (1-p)}{W^2}$$

$$n = \frac{(Za/\,_2)^2\, p\, (1-p)}{W^2} = \frac{(1.96)^2 (0.312)\, (0.688)}{(0.05)^2} = 329.8$$

By considering 10% non-response rate=32.98. Then the required sample size was 363.

Sampling techniques

Stratified sampling was undertaken to select the study of health institutions. All seven public health institutions in Awabale District were included and the sample size was proportionally allocated based on the number of target mothers. All eligible and consenting women attending for antenatal care during the study period were taken into the study consecutively until the sample size was reached.

Study variables

Dependent variable: depression during pregnancy (present/absent)

Independent variables

Socio-demographic factors: maternal age, educational status, marital status, occupation of the mother and family, monthly income

Obstetric factors: Gravidity, gestational age, history of abortion, unplanned pregnancy

Psycho-social factors: social support, relationship with a partner, husband support, violence

Clinical factors: a self-history of depression, family history of depression, history of chronic illness

Operational definitions

Antenatal depression: it is an illness in which pregnant women have an Edinburgh Postnatal Depression Scale (EPDS) score of 13 and above[21, 22]

Social support: Is support from the community ranges from a score of 3 to 14 according to the OSLO social support scale. A score of 3-8 is poor support, 9-11 is moderate support, and 12-14 is strong support.

Data collection procedure and data quality control

To assure the data quality, data were collected with face-to-face interviews by attained BSc Midwife in each institution after one-day data collection training was given to them together with two MSc holder midwife supervisors. The questionnaire was structured

and pre-tested which was first prepared in English and translated to local (Amharic) language and then again translated back to English by language experts. A pretest was conducted on 18 pregnant women of the sample size in Debre-Elias woreda and the necessary correction on the tool was employed accordingly.

Data collection and measurement tool

EPDS was administered to detect symptoms of depression and their socio-demographic data along with obstetric and psychosocial factors. EPDS is a 10 item questionnaire scored from zero up to three (higher score indicating more depressive symptoms), that has been validated for detecting depression in antepartum and postpartum samples in many countries. The instrument was validated in public health centers in Addis Ababa for postpartum use and showed a sensitivity of 84.6 % and specificity of 77.0 % at the cutoff score of 7/8[22]. Those pregnant women who score 13 and above were categorized as depressed women while pregnant women who scored below 13 were considered as non-depressed women [21, 22].

The OSLO-3 item social support scale was used to measure social support for pregnant women. Partners feeling on the current pregnancy can be defined as the sensation of pregnant women about the feeling of their partners concerning the current pregnancy. It was measured by asking whether her partner feeling on the current pregnancy was good or poor. Similarly, husband/partner support was assessed by asking women emotions about their partner's support to the health of the fetus and continuation of pregnancy. A structured Amharic version questioner containing socio-demographic characteristics, obstetric history, and psychosocial history, history of clinical factors, history of violence, and history of substance abuse was administered.

Data analysis technique

EpiData version 3.1 software was used for data entry and SPSS version 20 was used for analysis. Bivariate logistic regression was employed to identify an association between independent and dependent variables. Variables having a P-value of less than 0.2 in the bivariate logistic regression analysis were fitted into the multivariable logistic regression model to manage confounders. The 95% confidence interval of odds ratio

was computed and a variable having P-value less than 0.05 in the multivariable logistic regression analysis was considered as statistically significant.

Ethical consideration

The study was approved by the Institutional Health Research Ethics Review Committee of Debre Markos University, College of health science. An official letter was written from Debre Markos University to the selected health institutions. The participants enrolled in the study were informed about the study objectives, expected outcomes, benefits, and the risks associated with it. Written consent was taken from the participants before the interview.

Result

Socio-demographic factors

Three hundred fifty-four study participants gave the response to the questionnaire, giving a response rate of 97.5. %. The majorities of the respondents were Amhara 351 (99.2 %) and orthodox by religion were 293 (82.8 %). Three hundred thirty-five (94.6 %) of the women were married and 150(44.8%) were farmers in their occupation (Table 1).

Obstetric and clinical characteristics

Two hundred nine (59%) of the respondents had a history of pregnancy. Around 58(16.4%) and 9 (2.5%) of the respondents had a previous history of depression and a family history of depression respectively and 38(10.7%) had a history of chronic illness (Table 2).

Psychosocial, History of violence and substance use

more than Three-fourths (77.4%) of the respondents reported their husbands are happy with the occurrence of current pregnancy and a half (50.8%) of women explored good baby father support to the health of the fetus and continuation of pregnancy. Most

33(47.1%) and 249(70.3%) of the participants complained health-related problems were the most frequent emotionally disturbing factor in their lifetime and during the current pregnancy respectively. According to the OSLO social support scale, 112(31.6%) women have poor, 160(45.2%) moderate, and 82(23.2%) strong social support. Seventy-three (20.6%) of women's had a history of violence in their lifetime (Table 3).

Prevalence of antenatal depression

About 63 (17.8%) of respondents had antenatal depression (EPDS score ≥13). More than half (57%) of the respondents were able to laugh and see the funny side of things. On the other hand, (58.5%) of pregnant women felt sad or miserable most of the time (Table4)

Factors associated with antenatal depression

Age, marital status, educational status of the respondent, occupation of respondents and partners, history of abortion and stillbirth, trimester, unplanned pregnancy week at first ANC, poor husband support and feelings, social support, emotionally disturbing factors, history of depression, history of violence and substance use were significant factors in the bivariate regression analysis.

In the multivariable regression analysis; occupational status of the respondent [AOR=0.15(95%CI 0.001-0.25)], women educational status of high school and above[AOR=16.23(95%CI 2.46-107.27)], poor husband feeling on the current pregnancy[AOR=4.86(95%CI: 1.74-13.58)] and the previous history of depression [AOR=7.26(95%CI: 2.52-20.93)] were significant factors for antenatal depression (Table- 5).

Discussion

This study aimed to assess the prevalence of depression and associated factors among pregnant women attending antenatal care in public health institutions of Awabale Woreda, East Gojam zone, Amhara National Regional State, Northwestern Ethiopia, during March-April 2018. The study finding of antenatal depression (17.8%) in the current study was in line with similar reports in Bangladesh (18%) and Ethiopia (19.9%)

[23, 24]. The finding of this research was lower than the studies done in China (28.5%) [7], 29.9% and 31.2% in Ethiopia [21, 25], 33.8 in Tanzania[17], 38.5% and 47% in South Africa[17, 26]. This difference might be due to the difference in their population demographic characteristics, study design, period, and the difference in their investigatory or diagnostic tools.

Prevalence of antenatal depression was associated with the occupation of the women, educational status, history of depression, and poor husband feeling in the current pregnancy. Women who were running employees were 85% reduced to develop antenatal depression than housewives [AOR=0.15(0.001-0.25)]. This might be due to that, those who are employees may have social relationships and may have the satisfaction that makes the women economically independent [27]. Also, those housewife women are expending most of their time at home and alone. This loneliness may put them in depression [28].

Women with high school and above educational levels were 18 times more likely to develop antenatal depression than women who had no formal education [16.23(95%CI 2.46-107.27)]. This might be due to their difficulty in managing interpersonal relationship strains related to academic performance pressure and inability to translate their additional education into better mental health outcomes[29].On the other hand, different studies in different countries report the association of lower educational status with an increased prevalence of antenatal depression[23, 27, 30].

Those women who had a history of depression had 7.26 times the odds of developing antenatal depression than women who had no history of depression [AOR=7.26(95%CI: 2.52- 20.93)]. The comparable association was also reported from studies conducted in developing and developed countries [21]. This might be due to physical and hormonal changes occurring during pregnancy and the recurrence of depressive symptoms[27]. Conversely, the personal history of previous psychiatric illness was not found to be a significant risk factor for antenatal depression in a study conducted in Lahore, Pakistan [31].

Pregnant women who had poor husband feeling on the current pregnancy were 4.86 times more likely to develop antenatal depression as compared with good husband feeling towards current pregnancy [AOR=4.86(95%CI: 1.74-13.58)]. This is possible

because those partners who had good feelings about the pregnancy authorize the women on their home responsibilities and help women to have cared for their health and the health of the fetus. It might be also due to the effect of a poor husband's feeling on diminishing partner support[21].

The finding of this study shows no significant association between partner occupational status, trimester, unplanned pregnancy, social support, history of violence, and substance use in the multivariable model. This result seems consistent with other findings[21, 32]. On the other hand, contrary to this finding, those women who had a history of substance use had a higher risk of developing antenatal depression[17].

Limitations of the study

We cannot be certain that individuals with an EPDS score ≥ of 13 had depressive illness without confirmation. Concluding about partner feeling on the current pregnancy by asking pregnant women was an indirect conclusion and may have a biased result. Social desirability bias due to face-to-face interviews and using cross-sectional studies which do not show causality is also the limitation of this study.

Strength of the study

The main strength of this study was the data source. It used a primary data source during data collection.

Conclusion

This study showed that 17.8% of pregnant women develop antenatal depression. These notify a higher prevalence of antenatal depression in women attending antenatal care services at public health institutions. Women's occupational statuses, educational status, previous history of depression, and poor husband feeling on the current pregnancy were significant factors for antenatal depression.

Abbreviations

AOR: Adjusted Odds Ratio; CI: Confidence Interval; COR: Crude Odds Ratio; EPDS Edinburgh Postnatal Dépression Scale

Availability of data and materials

The data that support the findings of this study are available, but some restrictions may apply to the availability of these data as there are some sensitive issues. However, data are available from the corresponding authors upon reasonable request.

Competing interests

The authors declare that they have no competing interests.

Funding

Debre Markos University

Authors' contributions

All authors involved equally, read and approved the final manuscript.

Acknowledgments

We would like to express our deepest heartfelt thanks to Debre Markos University for their permission to do this research and we gratefully acknowledge all study individuals for their participation in the study.

Reference

1. **Pregnancy | definition of pregnancy by Medical dictionary**
2. Mirsanjari MO, Muda WAM, Ahmad A, Othman MS, Mirsanjari MM: **Depression symptoms in the second and third trimester of gestation**. 2012.
3. Pereira PK, Lovisi GM, Pilowsky DL, Lima LA, Legay LF: **Depression during pregnancy: prevalence and risk factors among women attending a public**

health clinic in Rio de Janeiro, Brazil. *Cadernos de Saúde Pública* 2009, **25**(12):2725-2736.

4. **Depressive Disorders | Psychology Today**

5. Getinet W, Amare T, Boru B, Shumet S, Worku W, Azale T: **Prevalence and Risk Factors for Antenatal Depression in Ethiopia: Systematic Review**. *Depression Research and Treatment* 2018, **2018**:3649269.
6. Zegeye A, Alebel A, Gebrie A, Tesfaye B, Belay YA, Adane F, Abie W: **Prevalence and determinants of antenatal depression among pregnant women in Ethiopia: a systematic review and meta-analysis**. *BMC Pregnancy and Childbirth* 2018, **18**(1):462.
7. Zeng Y, Cui Y, Li J: **Prevalence and predictors of antenatal depressive symptoms among Chinese women in their third trimester: a cross-sectional survey**. *BMC psychiatry* 2015, **15**(1):66.
8. **Antenatal depression**. *Black Dog Institute*
9. Alder J, Fink N, Bitzer J, Hösli I, Holzgreve W: **Depression and anxiety during pregnancy: a risk factor for obstetric, fetal and neonatal outcome? A critical review of the literature**. *The journal of maternal-fetal & neonatal medicine : the official journal of the European Association of Perinatal Medicine, the Federation of Asia and Oceania Perinatal Societies, the International Society of Perinatal Obstet* 2007, **20**(3):189-209.
10. Husain N, Munshi T, Jafri F, Husain M, Parveen A, Saeed Q, Tomenson B, Naeem F, Chaudhry N: **Antenatal Depression is Not Associated with Low-Birth Weight: A Study from Urban Pakistan**. *Frontiers in psychiatry* 2014, **5**:175.
11. Grote NK, Bridge JA, Gavin AR, Melville JL, Iyengar S, Katon WJ: **A meta-analysis of depression during pregnancy and the risk of preterm birth, low birth weight, and intrauterine growth restriction**. *Archives of general psychiatry* 2010, **67**(10):1012-1024.
12. Bonari L, Pinto N, Ahn E, Einarson A, Steiner M, Koren G: **Perinatal risks of untreated depression during pregnancy**. *Canadian journal of psychiatry Revue canadienne de psychiatrie* 2004, **49**(11):726-735.
13. Dunkel Schetter C, Tanner L: **Anxiety, depression and stress in pregnancy: implications for mothers, children, research, and practice**. *Curr Opin Psychiatry* 2012, **25**(2):141-148.
14. Pereira PK, Lovisi GM, Lima LA, Legay LF, de Cintra Santos JF, Santos SA, Thiengo DL, Valencia E: **Depression during pregnancy: review of epidemiological and clinical aspects in developed and developing countries**. In: *Psychiatric Disorders-Trends and Developments.* edn.: InTech; 2011.

15. Bisetegn TA, Mihretie G, Muche T: **Prevalence and Predictors of Depression among Pregnant Women in Debretabor Town, Northwest Ethiopia**. *PloS one* 2016, **11**(9):e0161108.
16. Sahile MA, Segni MT, Awoke T, Bekele D: **Prevalence and predictors of antenatal depressive symptoms among women attending Adama Hospital Antenatal Clinic, Adama, Ethiopia**. *International Journal of Nursing and Midwifery* 2017, **9**(5):58-64.
17. Manikkam L, Burns JK: **Antenatal depression and its risk factors: an urban prevalence study in KwaZulu-Natal**. *SAMJ: South African Medical Journal* 2012, **102**(12):940-944.
18. **Federal Democratic Republic of Ethiopia Ministry of Health.National MentalHealth Strategy 2012/13 - 2015/16.** 2012/13-2015/16.
19. Hanlon C, Alem A, Lund C, Hailemariam D, Assefa E, Giorgis TW, Chisholm D: **Moving towards universal health coverage for mental disorders in Ethiopia**. *International journal of mental health systems* 2019, **13**(1):1-16.
20. **Annual report of Awabale District health office** 2010 E.C.
21. Biratu A, Haile D: **Prevalence of antenatal depression and associated factors among pregnant women in Addis Ababa, Ethiopia: a cross-sectional study**. *Reproductive health* 2015, **12**(1):99.
22. Tesfaye M, Hanlon C, Wondimagegn D, Alem A: **Detecting postnatal common mental disorders in Addis Ababa, Ethiopia: validation of the Edinburgh postnatal depression scale and Kessler scales**. *Journal of affective disorders* 2010, **122**(1):102-108.
23. Nasreen HE, Edhborg M, Forsell Y, Kabir ZN: **Prevalence and associated factors of depressive and anxiety symptoms during pregnancy: a population based study in rural Bangladesh**. *BMC women's health* 2011, **11**(1):22.
24. Dibaba Y, Fantahun M, Hindin MJ: **The association of unwanted pregnancy and social support with depressive symptoms in pregnancy: evidence from rural Southwestern Ethiopia**. *BMC pregnancy and childbirth* 2013, **13**(1):135.
25. Martha Assefa Sahile1 MTS, Tadese Awoke3 and Dessalegn Bekele4: **Prevalence and predictors of antenatal depressive symptoms among women attending Adama Hospital Antenatal Clinic, Adama, Ethiopia**. *International Journal of Nursing and Midwifery*.
26. Rochat TJ, Tomlinson M, Bärnighausen T, Newell M-L, Stein A: **The prevalence and clinical presentation of antenatal depression in rural South Africa**. *Journal of Affective Disorders* 2011, **135**(1):362-373.
27. Yanikkerem E, Ay S, Mutlu S, Goker A: **Antenatal depression: prevalence and risk factors in a hospital based Turkish sample**. *J Pak Med Assoc* 2013, **63**(4):472-477.

28. Singh A, Misra N: **Loneliness, depression and sociability in old age**. *Industrial psychiatry journal* 2009, **18**(1):51.

29. Sarah K. Dixon SERK: **Depression and College Stress Among University Undergraduates: Do Mattering and Self-Esteem Make a Difference?**

30. Marcus SM, Flynn HA, Blow FC, Barry KL: **Depressive symptoms among pregnant women screened in obstetrics settings**. *Journal of Women's Health* 2003, **12**(4):373-380.

31. Humayun A, Haider I, Imran N, Iqbal H, Humayun N: **Antenatal depression and its predictors in Lahore, Pakistan/Depression prenatale et facteurs predictifs a Lahore (Pakistan)**. *Eastern Mediterranean Health Journal* 2013, **19**(4):327.

32. Hartley M, Tomlinson M, Greco E, Comulada WS, Stewart J, Le Roux I, Mbewu N, Rotheram-Borus MJ: **Depressed mood in pregnancy: prevalence and correlates in two Cape Town peri-urban settlements**. *Reproductive health* 2011, **8**(1):9.

Table 1: Socio-demographic characteristics of pregnant women attending ANC in Awabale district, East Gojjam zone, Northwest Ethiopia 2018(n=354)

Variables		Frequency	Percentage
**Maternal Age**	≤ 19	17	4.8
	20-34	262	74.0
	≥35	75	21.2
**Religion**	Orthodox Christian	293	82.8
	Muslim	61	17.2
**Ethnicity**	Amhara	351	99.2
	Oromo	3	0.8
**Marital status**	Not married	19	5.4
	Married	335	94.6
**Educational status of the respondent**	Illiterate(Unable to read and write)	170	48.0
	Elementary school (grade 1-8)	91	25.7
	High school and above	93	26.3
**Occupation of the respondent**	Employed	69	19.5
	Running personal business	60	16.9
	Housewife	225	63.6
**Educational status of the husband**	Illiterate(Unable to read and write)	187	55.8
	Elementary school (grade 1-8)	57	17.0
	High school and above	91	27.2
**Occupation of the husband**	Employed	77	23.0
	Running personal business	108	32.2
	Farmer	150	44.8
**Residence**	Rural	189	53.4
	Urban	165	46.6
**Monthly income**	≤ 500	45	12.7
	>500	309	87.3

Table 2: Obstetrics and clinical characteristics of pregnant women attending ANC in Awabale district, East Gojjam zone, Northwest Ethiopia 2018(n=354)

Variables		Frequency	Percentage
History of previous pregnancy	No	145	41.0
	Yes	209	59.0
Number of pregnancies	Primigravidas	145	41.0
	Multigravida	209	59.0
Previous history of abortion	N0	167	79.9
	Yes	42	20.1
Type of abortion	Spontaneous	37	88.1
	Induced	5	11.9
History of stillbirth	No	186	89.0
	Yes	23	11.0
Is the current pregnancy planned	No	48	13.6
	Yes	306	86.4
	First trimester	78	22.0
Trimester	Second trimester	195	55.1
	Third trimester	81	22.9
Gestational age at first ANC	Before 16 weeks	254	71.8
	After 16 weeks	100	28.2
Previous history of depression	No	296	83.6
	Yes	58	16.4
Family history of a depressive episode	No	345	97.5
	Yes	9	2.5
History of chronic illness	No	316	89.3
	Yes	38	10.7

Table 3: Psychosocial, history of violence and substance use of pregnant women attending ANC in Awabale district, East Gojjam zone, Northwest Ethiopia 2018(n=354)

Variables		Frequency	Percentage%
Husband feeling on the current pregnancy	Happy(Good)	274	77.4
	Not happy(Poor)	80	22.6
Husband support to the health of the fetus and continuation of pregnancy	Very good	148	41.8
	Good	180	50.8
	Not good	26	7.3
Social support(Based on OSLO social support scale)	Poor	112	31.6
	Moderate	160	45.2
	Strong	80	23.2
The emotionally disturbing factor for the last twelve month	No	284	80.2
	Yes	70	19.8
Psychosocial problems addressed at the antenatal clinic	No	29	8.2
	Yes	325	91.8
Enough information about the pregnancy and expected labor at ANC	No	23	6.5
	Yes	331	93.5
History of violence	No	281	79.4
	Yes	73	20.6
Lifetime history of substance use	No	196	55.4
	Yes	158	44.6
History of substance use during the current pregnancy	No	200	56.5
	Yes	154	43.5

Table 4: EPDS measurement of antenatal depression among pregnant women attending ANC in Awabale district, East Gojjam zone, Northwest Ethiopia 2018(n=354)

Variables		Frequency	Percentage
I have been able to laugh and see the funny side of things:	As much as I always could	203	57.3
	Not quite as much now	110	31.1
	Not so much now	24	6.8
	Not at all	17	4.8
I have look forward with enjoyment to things:	As much as I ever did	204	57.6
	Rather less than I used to	99	28.0
	Less than I used to	28	7.9
	Hardly at all	23	6.5
I have blamed myself unnecessarily when things went wrong:	Yes, most of the time	111	31.4
	Yes, some of the time	103	29.1
	Not very often	128	36.2
	No, never	12	3.4
I have been anxious or worried for no good reason:	No, not at all	133	37.6
	Hardly ever	82	23.2
	Yes, sometimes	125	35.3
	Yes, very often	14	4.0
I have felt scared or panicky for no very good reason:	Yes, quite a lot	88	24.9
	Yes, sometimes	112	31.6
	No, not much	118	33.3
	No, not at all	36	10.2
Things have been getting on top of me:	Yes, most of the time I haven't been able to cope at all	105	29.7
	Yes, sometimes I haven't been coping as well as usual	135	38.1
	No, most of the time I have coped quite well	108	30.5
	No, I have been coping	6	1.7
I have been so unhappy that I have had difficulty sleeping:	Yes, most of the time	139	39.3
	Yes, sometimes	118	33.3
	Not very often	68	19.2
	No, not at all	29	8.2
I have felt sad or miserable:	Yes, most of the time	207	58.5
	Yes, quite often	103	29.1
	Not very often	31	8.8
	No, not at all	13	3.7
I have so unhappy that I have been crying	Yes, most of the time	282	79.7
	Yes quite often	53	15
	Only occasionally	18	5.1
	No, never	1	0.3
The thought of harming myself has occurred to me:	Yes, quite often	0	0
	Sometimes	340	96.0
	Hardly ever	12	3.4
	Never	2	0.6

Table 5: Factors associated with antenatal depression among pregnant women attending ANC in Awabale district, East Gojjam zone, Northwest Ethiopia 2018(n=354)

variables		Depression		COR(95%CI)	AOR(95%CI)
		No	Yes		
Occupational status of the respondent	Employed	63	6	0.37 (0.15, 0.91)	0.15(0.001, 0.25)
	Running personal business	49	11	0.87(0.42, 1.81)	0.48(0.07, 3.21)
	Housewife	179	46	1	1
Educational status of the respondent	Illiterate	147	23	1	1
	Elementary school	64	27	1.68(0.82, 3.45)	1.08(0.18, 6.4)
	High school and above	80	13	1.04(0.5, 2.16)	16.23(2.46, 107.27)
Occupational status of the husband	Employed	66	11	0.57(0.27, 1.2)	1.47(0.21, 10.04)
	Running personal business	102	6	0.20(0.81, 0.5)	0.22(0.044, 1.09)
	Farmer	116	34	1	1
Trimester	First	88	10	2.4(1.30, 4.42)	1.90(0.47, 7.74)
	Second	154	41	1	1
	Third	69	12	0.65(0.32, 1.32)	0.87(0.28, 3.69)
Planned pregnancy	No	24	24	6.85(3.55,13.22)	3.06(0.73, 12.87)
	Yes	267	39	1	1
Social support	Poor	81	31	2.40(1.30, 4.42)	1.14(0.28, 4.61)
	Moderate	138	22	1	1
	Strong	72	10	0.87(0.39, 1.94)	0.92(0.24, 3.61)
Husband feeling on the current pregnancy	Good	248	26	1	1
	Poor	43	37	8.21(4.52, 4.91)	4.86(1.74, 13.58)
Previous history of depression	No	256	40	1	1
	Yes	35	23	4.21(2.26, 7.84)	7.26(2.52, 20.93)
History of violence	No	247	34	1	1
	Yes	44	29	4.79(2.65, 8.64)	1.27(0.34, 4.79)
Lifetime history of substance use	No	116	42	1	1
	Yes	175	21	3.02(1.70, 5.36)	1.88(0.73, 4.84)
History of substance use in the current pregnancy	No	114	40	1	1
	Yes	177	23	2.70(1.54, 4.75)	0.09(0.004, 1.82)

Prevalence of Depression and Associated Factors among Pregnant Women Attending Antenatal Care in Public Health Institutions of Awabale Woreda, East Gojjam Zone, Northwestern Ethiopia: a cross-sectional study

Alemayehu Bantie[1], Getachew Mulu kassa[2], Haymanot Zeleke[2], Liknaw Bewket Zeleke[2], Bewket Yeserah Aynalem[2]*

[1] Debre Markos, Ethiopia

[2] Health Science College, Debre Markos University, Debre Markos, Ethiopia

***Corresponding author:**

Email: by123bewket@gmail.com

Abstract

Background: Antenatal depression is a serious health problem and has negative consequences for the mother, fetus, and the entire family. However, it is a neglected component of care for women in pregnancy. The purpose of this study was to assess the prevalence of depression and associated factors among pregnant women attending antenatal clinics in public health institutions, in the Awabale Woreda

Method: An institutional-based cross-sectional study was conducted in 2018 and a stratified sampling technique was used to select the study health institutions. A structured questionnaire was used to collect data. Edinburgh Postnatal Depression Scale was used to declare the presence of antenatal depression with a cut point score of 13 and above.

Result: This study showed that 63(17.8%) pregnant mothers had antenatal depression. Women who were running their business were 85% reduced to develop antenatal depression than housewives [AOR=0.15(0.001-0.25)]. Pregnant women had high school and above educational level 18 times higher odds of developing antenatal depression than women who had no formal education [AOR18.15 (2.73-120.76)].

Women who had poor husband feeling on the current pregnancy were 4.94 more likely to develop antenatal depression than women who had good partner feeling on the current pregnancy [AOR=4.94(95%CI: 1.78-13.72)]. Women who had a history of depression were 8.2 times to develop antenatal depression than women who had no history of depression [AOR=8.22 (95%CI: 2.87-23.57)].

Conclusion: This study revealed that one-fifth of pregnant women developed antenatal depression. Women's occupational status, educational status, previous history of depression, and poor husband feeling on the current pregnancy were the significant factors of antenatal depression.

Keywords: Depression; Antenatal depression; Antenatal care; Awabel Woreda

Introduction

Pregnancy is the period from the fertilization of the egg by sperm to the delivery of the fetus and usually lasts 40 weeks. Starting from the last normal menstrual period, it is divided into three trimesters, each lasting three months[1]. It is a distinctive social and biological event in a woman's life [2, 3].

A depressive disorder is an illness that involves the body, mood, and thoughts in which the person has unrelenting sentiments of unhappiness and irrelevance and a lack of aspiration to engage in previously enjoyable activities that last more than two weeks. When these sentiments last for a short period, it may be a case of feeling sadness[4]. It is the most frequent expressive disorder in women and the general population, and one in every five people has depression with more than the twofold increased incidence in women than men [2]. Psychiatric history, stressful life events, lack of social support, teenage pregnancy, low educational level, low income, and violence against women are the factors that make depression prevalent in pregnant women [3, 5, 6].

Antenatal depression means depression that starts from conception to delivery[7]. Pregnant women experience symptoms similar to general depression and may interfere with their normal day-to-day activities. It may occur at any stage of pregnancy and can be a reaction to the pregnancy itself, due to health issues, major life stresses, genetic

and biochemical basis, or due to a continuation or relapse of a pre-pregnancy condition[8]. Antenatal depression ends with poor fetal, infant development, and maternal outcomes like small for gestational age, prematurity, intrauterine growth problem; postnatal depression preeclampsia, anemia; educational problem, malnutrition, respiratory disorders, and mental retardation [9-13].

Most of the researches was undertake on postnatal depression. The majority of studies on the prevalence of antenatal depression and associated factors have been conducted in developed countries [14]. However, there is a paucity of studies, which examined the prevalence and associated factors of antenatal depression in low-income countries like Ethiopia. There are studies undertaken on antenatal depression. Yet, they focus on adolescent pregnant women and women's that have antenatal care service in hospital settings[3]. Those studies have a limitation on addressing pregnant women in all age groups. In addition, they are likely to have a bias because most women with hindrance in their pregnancies are likely to seek antenatal care services in hospitals. There are studies, which were excluded illiterate women from the study[15]. This may lead to selection bias because educational status has a significant association with antenatal depression as described in the literature.

The Federal Democratic Republic of Ethiopia National Mental Health Strategy promotes a decentralized approach in which mental health services are to be offered from local health institutions up to tertiary hospitals to ensure access to treatment near to their living area and with a less restraining setting [16]. This study aimed to assess the prevalence of depression and associated factors among pregnant women attending antenatal clinics in public health institutions, in Awabale Woreda.

Methods

Study area and period

The study was conducted in 2018 in Awabale woreda which is found in the Amhara region, North West Ethiopia, which is located 300 km from Addis Ababa, the capital city of Ethiopia, and located 265 km from Bihar Dar, the capital city of the Amhara region. The population of the Woreda is 146260, of which 37413 are females aged 15-49

years[17]. There are seven public health institutions (one primary hospital and six health centers) eligible for this study in Awabale Woreda. Maternal health services are free of charge in Ethiopia at public health institutions including public health institutions included in this study. Antenatal care is provided in all public health institutions of Awabale. The Woreda has an annual ANC plan of 5590(1309 in Lumame health center, 648 in Shebela health center, 641 in Wejele health center, 665 in Lega health center, 901 in Yesenbet health center, 765 in Tsid-Maryam health center and 661 in Lumame primary hospital)[17]. This study was conducted in public health institutions of AwabaleWoreda, in 2018.

Study design

An institutional-based cross-sectional study design was employed.

Study participants

All pregnant women attending antenatal care in Awabale Woreda public health institutions were the source population. All pregnant women attending antenatal care in selected public health institutions during the study period were the study population. Pregnant women at any age of gestation who come to the selected health institutions for antenatal care visits during the study period were included in the study and we excluded those who were critically ill during the data collection period.

Sample size

The required sample size was calculated using a single population proportion formula with the assumption of 31.2 % of depression during pregnancy from a study conducted in Adama, Ethiopia[18] and 5% marginal error (w), a standard Z score of 1.96 corresponding to a 95% confidence interval ($Z_{a/2}$),and 10% non-response rate.

$$n = \frac{(Za/_2)^2\, p\, (1-p)}{W^2}$$

$$n = \frac{(Za/_2)^2\, p\, (1-p)}{W^2} = \frac{(1.96)^2 (0.312)\, (0.688)}{(0.05)^2} = 329.8$$

By considering 10% non response rate=32.98. Then the required sample size was 363.

Sampling techniques

Stratified sampling was undertaken to select the study of health institutions. All seven public health institutions in Awabale District were included and the sample size was proportionally allocated based on the number of target mothers. All eligible and consenting women attending for antenatal care during the study period were taken into the study consecutively until the sample size was reached.

Study variables

Dependent variable: depression during pregnancy (present/absent)

Independent variables

Socio-demographic factors: maternal age, educational status, marital status, occupation of the mother and family, monthly income

Obstetric factors: Gravidity, gestational age, history of abortion, unplanned pregnancy

Psycho-social factors: social support, relationship with a partner, husband support, violence

Clinical factors: a self history of depression, family history of depression, history of chronic illness

Operational definitions

Antenatal depression: it is an illness in which pregnant women have an Edinburgh Postnatal Depression Scale (EPDS) score of 13 and above[19, 20]

Social support: Is support from the community ranges from a score of 3 to 14 according to the OSLO social support scale. A score of 3-8 is poor support, 9-11 is moderate support, and 12-14 is strong support.

Data collection procedure and data quality control

To assure the data quality, data were collected with face-to-face interviews by attained BSc Midwife in each institution after one-day data collection training was given to them together with two MSc holder supervisors. The questionnaire was structured and pre-

tested which was first prepared in English and translated to local (Amharic) language and then again translated back to English. A pretest was conducted on 18 pregnant women of the sample size other than the study area and the necessary correction on the tool was employed accordingly.

Data collection and measurement tool

EPDS was administered to detect symptoms of depression and their socio-demographic data along with obstetric and psychosocial factors. EPDS is a 10 item questionnaire scored from zero up to three (higher score indicating more depressive symptoms), that has been validated for detecting depression in antepartum and postpartum samples in many countries. The instrument was validated in public health centers in Addis Ababa for postpartum use and showed a sensitivity of 84.6 % and specificity of 77.0 % at the cutoff score of 7/8[20]. Those pregnant women who score 13 and above were categorized as depressed women while pregnant women who scored below 13 were considered as non-depressed women.

The OSLO-3 item social support scale was used to measure social support for pregnant women. Partners feeling on the current pregnancy can be defined as the sensation of pregnant women about the feeling of their partners concerning the current pregnancy. It was measured by asking whether her partner feeling on the current pregnancy was good or poor. Similarly, husband/partner support was assessed by asking women emotions about their partner's support to the health of the fetus and continuation of pregnancy. A structured Amharic version questioner containing socio-demographic characteristics, obstetric history, and psychosocial history, history of clinical factors, history of violence, and history of substance abuse was administered.

Data analysis technique

EpiData version 3.1 software was used for data entry and SPSS version 20 was used for analysis. Bivariate logistic regression was employed to identify an association between independent and dependent variables. Variables having a P-value of less than 0.2 in the bivariate logistic regression analysis were fitted into the multivariable logistic regression model to manage confounders. The 95% confidence interval of odds ratio

was computed and a variable having P-value less than 0.05 in the multivariable logistic regression analysis was considered as statistically significant. The result was presented by tables and text.

Ethical consideration

The study was approved by the Institutional Health Research Ethics Review Committee of Debre Markos University, College of health science. An official letter was written from Debre Markos University to the selected health institutions. The participants enrolled in the study were informed about the study objectives, expected outcomes, benefits, and the risks associated with it. Written consent was taken from the participants before the interview.

Result

Socio-demographic factors

Three hundred fifty-four study participants gave the response to the questionnaire, giving a response rate of 97.5. %. The majorities of the respondents were Amhara 351 (99.2 %) and orthodox by religion were 293 (82.8 %). Three hundred thirty-five (94.6 %) of the women were married and 150(44.8%) were farmers in their occupation (Table 1).

Obstetric and clinical characteristics

Two hundred nine (59%) of the respondents had a history of pregnancy. Around 58(16.4%) and 9 (2.5%) of the respondents had a previous history of depression and a family history of depression respectively and 38(10.7%) had a history of chronic illness (Table 2).

Psychosocial, History of violence and substance use

more than Three-fourths (77.4%) of the respondents reported their husbands are happy with the occurrence of current pregnancy and a half (50.8%) of women explored good

baby father support to the health of the fetus and continuation of pregnancy. Most 33(47.1%) and 249(70.3%) of the participants complained health-related problems were the most frequent emotionally disturbing factor in their lifetime and during the current pregnancy respectively. According to the OSLO social support scale, 112(31.6%) women have poor, 160(45.2%) moderate, and 82(23.2%) strong social support. Seventy-three (20.6%) of women's had a history of violence in their lifetime (Table 3).

Prevalence of antenatal depression

About 63 (17.8%) of respondents had antenatal depression (EPDS score ≥13). More than half (57%) of the respondents were able to laugh and see the funny side of things. On the other hand, (58.5%) of pregnant women felt sad or miserable most of the time (Table4)

Factors associated with antenatal depression

Age, marital status, educational status of the respondent, occupation of respondents and partners, history of abortion and stillbirth, trimester, unplanned pregnancy week at first ANC, poor husband support and feelings, social support, emotionally disturbing factors, history of depression, history of violence and substance use were significant factors in the bivariate regression analysis.

In the multivariable regression analysis; occupational status of the respondent [AOR=0.15(95%CI 0.001-0.25)], women educational status of high school and above[AOR=16.23(95%CI 2.46-107.27)], poor husband feeling on the current pregnancy[AOR=4.86(95%CI: 1.74-13.58)] and the previous history of depression [AOR=7.26(95%CI: 2.52-20.93)] were significant factors for antenatal depression (Table- 5).

Discussion

This study aimed to assess the prevalence of depression and associated factors among pregnant women attending antenatal care in public health institutions of Awabale Woreda, East Gojam zone, Amhara National Regional State, Northwestern Ethiopia, during March-April 2018. The study finding of antenatal depression (17.8%) in the

current study was in line with similar reports in Bangladesh (18%) and Ethiopia (19.9%) [21, 22]. The finding of this research was lower than the studies done in China (28.5%) [7], 29.9% and 31.2% in Ethiopia [19, 23], 33.8 in Tanzania[15], 38.5% and 47% in South Africa[15, 24]. This difference might be due to the difference in their population demographic characteristics, study design, period, and the difference in their investigatory or diagnostic tools.

Prevalence of antenatal depression was associated with the occupation of the women, educational status, history of depression, and poor husband feeling in the current pregnancy. Women who were running their business were 85% reduced to develop antenatal depression than housewives [AOR=0.15(0.001-0.25)]. This might be due to that, those who have their own business may have social relationships and putting women economically independent [25]. Also, those housewife women are expending most of their time at home and alone. This loneliness may put them in depression [26].

Women with high school and above educational levels were 18 times more likely to develop antenatal depression than women who had no formal education [16.23(95%CI 2.46-107.27)]. This might be due to their difficulty in managing interpersonal relationship strains related to academic performance pressure and inability to translate their additional education into better mental health outcomes[27].On the other hand, different studies in different countries report the association of lower educational status with an increased prevalence of antenatal depression[21, 25, 28].

Those women who had a history of depression had 7.26 times the odds of developing antenatal depression than women who had no history of depression [AOR=7.26(95%CI: 2.52- 20.93)]. The comparable association was also reported from studies conducted in developing and developed countries [19]. This might be due to physical and hormonal changes occurring during pregnancy and the recurrence of depressive symptoms[25]. Conversely, the personal history of previous psychiatric illness was not found to be a significant risk factor for antenatal depression in a study conducted in Lahore, Pakistan [29].

Pregnant women who had poor husband feeling on the current pregnancy were 4.86 times more likely to develop antenatal depression as compared with good husband feeling towards current pregnancy [AOR=4.86(95%CI: 1.74-13.58)]. This is possible

because those partners who had good feelings about the pregnancy authorize the women on their home responsibilities and help women to have cared for their health and the health of the fetus. It might be also due to the effect of a poor husband's feeling on diminishing partner support[19].

The finding of this study shows no significant association between partner occupational status, trimester, unplanned pregnancy, social support, history of violence, and substance use in the multivariable model. This result seems consistent with other findings[19, 30]. On the other hand, contrary to this finding, those women who had a history of substance use had a higher risk of developing antenatal depression[15].

Limitations of the study

We cannot be certain that individuals with an EPDS score ≥ of 13 had depressive illness without confirmation. Moreover, the EPDS cut-off score varies in different kinds of literature and this could be the reason for the different prevalence of antenatal depression in different kinds of literature. Concluding about partner feeling on the current pregnancy by asking pregnant women was an indirect conclusion and may have a biased result. Social desirability bias due to face-to-face interviews and using cross-sectional studies which do not show causality is also the limitation of this study.

Strength of the study

The main strength of this study was the data source. It used a primary data source during data collection.

Conclusion

This study showed that 17.8% of pregnant women develop antenatal depression. These notify a higher prevalence of antenatal depression in women attending antenatal care services at public health institutions. Women's occupational statuse, educational status, previous history of depression, and poor husband feeling on the current pregnancy were significant factors for antenatal depression.

Recommendation

This higher prevalence of antenatal depression should alert health care professionals for conscious screening of housewife pregnant women and pregnant women with the educational status of high school and above for all depressive symptoms by focusing on the previous history of depression as an overall health assessment. Encouraging male's involvement in maternal health service and identifying those partners who have poor feelings about the current pregnancy and providing appropriate counseling should be in the mind of all stakeholders. Enhancing employment opportunities and empowering housewife women's involvement in income-earning activities should be a matter of both governmental and non-governmental stakeholders.

Formulating strategies for better mental health outcomes and improving women's capability in translating their educational status into better mental health outcomes should be in the mind of both the Federal Ministry of health (FMO) and the Regional health bureau in collaboration with the Ministry of education. Expansion of mental health services far to the extreme rural areas and providing appropriate counseling, treatment, referral, and linkage of women with antenatal depression to the appropriate health institution should be considered by all stakeholders. Strengthening admission of pregnant women on early gestation to the nearby health center by focusing on those housewife women and women with a previous history of depression are the recommended interventions.

Abbreviations

AOR: Adjusted Odds Ratio; CI: Confidence Interval; COR: Crude Odds Ratio; EPDS Edinburgh Postnatal Dépression Scale

Availability of data and materials

The data that support the findings of this study are available, but some restrictions may apply to the availability of these data as there are some sensitive issues. However, data are available from the corresponding authors upon reasonable request.

Competing interests

The authors declare that they have no competing interests.

Funding

Debre Markos University

Authors’ contributions

All authors involved equally, read and approved the final manuscript.

Acknowledgments

We would like to express our deepest heartfelt thanks to Debre Markos University for their permission to do this research and we gratefully acknowledge all study individuals for their participation in the study.

Reference

1. **Pregnancy | definition of pregnancy by Medical dictionary**

https://medical-dictionarythefreedictionarycom/pregnancy.

2. Mirsanjari MO, Muda WAM, Ahmad A, Othman MS, Mirsanjari MM: **Depression symptoms in the second and third trimester of gestation**. 2012.

3. Pereira PK, Lovisi GM, Pilowsky DL, Lima LA, Legay LF: **Depression during pregnancy: prevalence and risk factors among women attending a public health clinic in Rio de Janeiro, Brazil**. *Cadernos de Saúde Pública* 2009, **25**(12):2725-2736.

4. **Depressive Disorders | Psychology Today**

https://wwwpsychologytodaycom/conditions/depressive-disorders

5. Getinet W, Amare T, Boru B, Shumet S, Worku W, Azale T: **Prevalence and Risk Factors for Antenatal Depression in Ethiopia: Systematic Review**. *Depression Research and Treatment* 2018, **2018**:3649269.

6. Zegeye A, Alebel A, Gebrie A, Tesfaye B, Belay YA, Adane F, Abie W: **Prevalence and determinants of antenatal depression among pregnant women in Ethiopia: a systematic review and meta-analysis**. *BMC Pregnancy and Childbirth* 2018, **18**(1):462.

7. Zeng Y, Cui Y, Li J: **Prevalence and predictors of antenatal depressive symptoms among Chinese women in their third trimester: a cross-sectional survey**. *BMC psychiatry* 2015, **15**(1):66.

8. **Antenatal depression**. *Black Dog Institute* http://wwwblackdoginstituteorgau/public/depression/inpregnancypostnatal/antenataldepressioncfm.

9. Alder J, Fink N, Bitzer J, Hösli I, Holzgreve W: **Depression and anxiety during pregnancy: a risk factor for obstetric, fetal and neonatal outcome? A critical review of the literature**. *The journal of maternal-fetal & neonatal medicine : the official journal of the European Association of Perinatal Medicine, the Federation of Asia and Oceania Perinatal Societies, the International Society of Perinatal Obstet* 2007, **20**(3):189-209.

10. Husain N, Munshi T, Jafri F, Husain M, Parveen A, Saeed Q, Tomenson B, Naeem F, Chaudhry N: **Antenatal Depression is Not Associated with Low-Birth Weight: A Study from Urban Pakistan**. *Frontiers in psychiatry* 2014, **5**:175.

11. Grote NK, Bridge JA, Gavin AR, Melville JL, Iyengar S, Katon WJ: **A meta-analysis of depression during pregnancy and the risk of preterm birth, low birth weight, and intrauterine growth restriction**. *Archives of general psychiatry* 2010, **67**(10):1012-1024.

12. Bonari L, Pinto N, Ahn E, Einarson A, Steiner M, Koren G: **Perinatal risks of untreated depression during pregnancy**. *Canadian journal of psychiatry Revue canadienne de psychiatrie* 2004, **49**(11):726-735.

13. Dunkel Schetter C, Tanner L: **Anxiety, depression and stress in pregnancy: implications for mothers, children, research, and practice**. *Curr Opin Psychiatry* 2012, **25**(2):141-148.

14. Pereira PK, Lovisi GM, Lima LA, Legay LF, de Cintra Santos JF, Santos SA, Thiengo DL, Valencia E: **Depression during pregnancy: review of epidemiological and clinical aspects in developed and developing countries**. In: *Psychiatric Disorders-Trends and Developments.* edn.: InTech; 2011.

15. Manikkam L, Burns JK: **Antenatal depression and its risk factors: an urban prevalence study in KwaZulu-Natal**. *SAMJ: South African Medical Journal* 2012, **102**(12):940-944.

16. **Federal Democratic Republic of Ethiopia Ministry of Health.National MentalHealth Strategy 2012/13 - 2015/16.** 2012/13-2015/16.

17. **Annual report of Awabale District health office** 2010 E.C.

18. **Assessment on Prevalence of Antenatal Depressive Disorders and Associated Factors amongAdama Hospital Antenatal Clinic Attendants,Adama, Ethiopia,2011.Unpublished study.** *Ethiopian Public Health Association* 2011.

19. Biratu A, Haile D: **Prevalence of antenatal depression and associated factors among pregnant women in Addis Ababa, Ethiopia: a cross-sectional study**. *Reproductive health* 2015, **12**(1):99.

20. Tesfaye M, Hanlon C, Wondimagegn D, Alem A: **Detecting postnatal common mental disorders in Addis Ababa, Ethiopia: validation of the Edinburgh postnatal depression scale and Kessler scales**. *Journal of affective disorders* 2010, **122**(1):102-108.

21. Nasreen HE, Edhborg M, Forsell Y, Kabir ZN: **Prevalence and associated factors of depressive and anxiety symptoms during pregnancy: a population based study in rural Bangladesh**. *BMC women's health* 2011, **11**(1):22.

22. Dibaba Y, Fantahun M, Hindin MJ: **The association of unwanted pregnancy and social support with depressive symptoms in pregnancy: evidence from rural Southwestern Ethiopia**. *BMC pregnancy and childbirth* 2013, **13**(1):135.

23. Martha Assefa Sahile1 MTS, Tadese Awoke3 and Dessalegn Bekele4: **Prevalence and predictors of antenatal depressive symptoms among women attending Adama Hospital Antenatal Clinic, Adama, Ethiopia**. *International Journal of Nursing and Midwifery*.

24. Rochat TJ, Tomlinson M, Bärnighausen T, Newell M-L, Stein A: **The prevalence and clinical presentation of antenatal depression in rural South Africa**. *Journal of Affective Disorders* 2011, **135**(1):362-373.

25. Yanikkerem E, Ay S, Mutlu S, Goker A: **Antenatal depression: prevalence and risk factors in a hospital based Turkish sample**. *J Pak Med Assoc* 2013, **63**(4):472-477.

26. Singh A, Misra N: **Loneliness, depression and sociability in old age**. *Industrial psychiatry journal* 2009, **18**(1):51.

27. Sarah K. Dixon SERK: **Depression and College Stress Among University Undergraduates: Do Mattering and Self-Esteem Make a Difference?**

28. Marcus SM, Flynn HA, Blow FC, Barry KL: **Depressive symptoms among pregnant women screened in obstetrics settings**. *Journal of Women's Health* 2003, **12**(4):373-380.

29. Humayun A, Haider I, Imran N, Iqbal H, Humayun N: **Antenatal depression and its predictors in Lahore, Pakistan/Depression prenatale et facteurs**

predictifs a Lahore (Pakistan). *Eastern Mediterranean Health Journal* 2013, **19**(4):327.

30. Hartley M, Tomlinson M, Greco E, Comulada WS, Stewart J, Le Roux I, Mbewu N, Rotheram-Borus MJ: **Depressed mood in pregnancy: prevalence and correlates in two Cape Town peri-urban settlements**. *Reproductive health* 2011, **8**(1):9.

Table 1: Socio-demographic characteristics of pregnant women attending ANC in Awabale district, East Gojjam zone, Northwest Ethiopia 2018(n=354)

Variables		Frequency	Percentage
**Maternal Age**	≤ 19	17	4.8
	20-34	262	74.0
	≥35	75	21.2
**Religion**	Orthodox Christian	293	82.8
	Muslim	61	17.2
**Ethnicity**	Amhara	351	99.2
	Oromo	3	0.8
**Marital status**	Not married	19	5.4
	Married	335	94.6
**Educational status of the respondent**	Illiterate(Unable to read and write)	170	48.0
	Elementary school (grade 1-8)	91	25.7
	High school and above	93	26.3
**Occupation of the respondent**	Employed	69	19.5
	Running personal business	60	16.9
	Housewife	225	63.6
**Educational status of the husband**	Illiterate(Unable to read and write)	187	55.8
	Elementary school (grade 1-8)	57	17.0
	High school and above	91	27.2
**Occupation of the husband**	Employed	77	23.0
	Running personal business	108	32.2
	Farmer	150	44.8
**Residence**	Rural	189	53.4
	Urban	165	46.6
**Monthly income**	≤ 500	45	12.7
	>500	309	87.3

Table 2: Obstetrics and clinical characteristics of pregnant women attending ANC in Awabale district, East Gojjam zone, Northwest Ethiopia 2018(n=354)

Variables		Frequency	Percentage
History of previous pregnancy	No	145	41.0
	Yes	209	59.0
Number of pregnancies	Primigravidas	145	41.0
	Multigravida	209	59.0
Previous history of abortion	N0	167	79.9
	Yes	42	20.1
Type of abortion	Spontaneous	37	88.1
	Induced	5	11.9
History of stillbirth	No	186	89.0
	Yes	23	11.0
Is the current pregnancy planned	No	48	13.6
	Yes	306	86.4
	First trimester	78	22.0
Trimester	Second trimester	195	55.1
	Third trimester	81	22.9
Gestational age at first ANC	Before 16 weeks	254	71.8
	After 16 weeks	100	28.2
Previous history of depression	No	296	83.6
	Yes	58	16.4
Family history of a depressive episode	No	345	97.5
	Yes	9	2.5
History of chronic illness	No	316	89.3
	Yes	38	10.7

Table 3: Psychosocial, history of violence and substance use of pregnant women attending ANC in Awabale district, East Gojjam zone, Northwest Ethiopia 2018(n=354)

**Variables**		**Frequency**	**Percentage%**
Husband feeling on the current pregnancy	Happy(Good)	274	77.4
	Not happy(Poor)	80	22.6
Husband support to the health of the fetus and continuation of pregnancy	Very good	148	41.8
	Good	180	50.8
	Not good	26	7.3
Social support(Based on OSLO social support scale)	Poor	112	31.6
	Moderate	160	45.2
	Strong	80	23.2
The emotionally disturbing factor for the last twelve month	No	284	80.2
	Yes	70	19.8
Psychosocial problems addressed at the antenatal clinic	No	29	8.2
	Yes	325	91.8
Enough information about the pregnancy and expected labor at ANC	No	23	6.5
	Yes	331	93.5
History of violence	No	281	79.4
	Yes	73	20.6
Lifetime history of substance use	No	196	55.4
	Yes	158	44.6
History of substance use during the current pregnancy	No	200	56.5
	Yes	154	43.5

Table 4: EPDS measurement of antenatal depression among pregnant women attending ANC in Awabale district, East Gojjam zone, Northwest Ethiopia 2018(n=354)

Variables		Frequency	Percentage
I have been able to laugh and see the funny side of things:	As much as I always could	203	57.3
	Not quite as much now	110	31.1
	Not so much now	24	6.8
	Not at all	17	4.8
I have look forward with enjoyment to things:	As much as I ever did	204	57.6
	Rather less than I used to	99	28.0
	Less than I used to	28	7.9
	Hardly at all	23	6.5
I have blamed myself unnecessarily when things went wrong:	Yes, most of the time	111	31.4
	Yes, some of the time	103	29.1
	Not very often	128	36.2
	No, never	12	3.4
I have been anxious or worried for no good reason:	No, not at all	133	37.6
	Hardly ever	82	23.2
	Yes, sometimes	125	35.3
	Yes, very often	14	4.0
I have felt scared or panicky for no very good reason:	Yes, quite a lot	88	24.9
	Yes, sometimes	112	31.6
	No, not much	118	33.3
	No, not at all	36	10.2
Things have been getting on top of me:	Yes, most of the time I haven't been able to cope at all	105	29.7
	Yes, sometimes I haven't been coping as well as usual	135	38.1
	No, most of the time I have coped quite well	108	30.5
	No, I have been coping	6	1.7
I have been so unhappy that I have had difficulty sleeping:	Yes, most of the time	139	39.3
	Yes, sometimes	118	33.3
	Not very often	68	19.2
	No, not at all	29	8.2
I have felt sad or miserable:	Yes, most of the time	207	58.5
	Yes, quite often	103	29.1
	Not very often	31	8.8
	No, not at all	13	3.7
I have so unhappy that I have been crying	Yes, most of the time	282	79.7
	Yes quite often	53	15
	Only occasionally	18	5.1
	No, never	1	0.3
The thought of harming myself has occurred to me:	Yes, quite often	0	0
	Sometimes	340	96.0
	Hardly ever	12	3.4
	Never	2	0.6

Table 5: Factors associated with antenatal depression among pregnant women attending ANC in Awabale district, East Gojjam zone, Northwest Ethiopia 2018(n=354)

variables		Depression		COR(95%CI)	AOR(95%CI)
		No	Yes		
Occupational status of the respondent	Employed	63	6	0.37 (0.15, 0.91)	0.16(0.001, 0.25)
	Running personal business	49	11	0.87(0.42, 1.81)	0.48(0.07, 3.21)
	Housewife	179	46	1	1
Educational status of the respondent	Illiterate	147	23	1	1
	Elementary school	64	27	1.68(0.82, 3.45)	1.08(0.18, 6.4)
	High school and above	80	13	1.04(0.5, 2.16)	16.23(2.46, 107.27)
Occupational status of the husband	Employed	66	11	0.57(0.27, 1.2)	1.47(0.21, 10.04)
	Running personal business	102	6	0.20(0.81, 0.5)	0.22(0.044, 1.09)
	Farmer	116	34	1	1
Trimester	First	88	10	2.4(1.30, 4.42)	1.90(0.47, 7.74)
	Second	154	41	1	1
	Third	69	12	0.65(0.32, 1.32)	0.87(0.28, 3.69)
Planned pregnancy	No	24	24	6.85(3.55,13.22)	3.06(0.73, 12.87)
	Yes	267	39	1	1
Social support	Poor	81	31	2.40(1.30, 4.42)	1.14(0.28, 4.61)
	Moderate	138	22	1	1
	Strong	72	10	0.87(0.39, 1.94)	0.92(0.24, 3.61)
Husband feeling on the current pregnancy	Good	248	26	1	1
	Poor	43	37	8.21(4.52, 4.91)	4.86(1.74, 13.58)
Previous history of depression	No	256	40	1	1
	Yes	35	23	4.21(2.26, 7.84)	7.26(2.52, 20.93)
History of violence	No	247	34	1	1
	Yes	44	29	4.79(2.65, 8.64)	1.27(0.34, 4.79)
Lifetime history of substance use	No	116	42	1	1
	Yes	175	21	3.02(1.70, 5.36)	1.88(0.73, 4.84)
History of substance use in the current pregnancy	No	114	40	1	1
	Yes	177	23	2.70(1.54, 4.75)	0.09(0.004, 1.82)

Dear reviewers

Thank you for your positive and constructive comments. I have provided responses based on your comments and questions.

Reviewer #1

1. **There is a paucity of data on antenatal depression in Ethiopia, yet there is a lot of work published from 2006 (Hanlon et al) to 2021.**

- ✓ **We have corrected it based on the given comment**

2. **Can you please acknowledge the source of this information - REFERENCES?**
   - ✓ This is our suggestion we have corrected it.
3. **What is the difference between your study site/population and that which has been reported in the literature already?**
   - ✓ The findings in the previous study were not consistent which shows further study was needed
   - ✓ The study population of our study area is different from other study populations like Adama and Addis Ababa and other places through lifestyle and perception about pregnancy and its importance.
4. **Ethically, women aged below 18 cannot consent. How did you cater to them?**
   - ✓ We took assent from their family if they came with them. If they came alone, we took assent from their health care providers
5. **The EPDS was validated in Ethiopia and a cut-off score of 7/8 was found to be satisfactory. Then why did you use a cut-off score of 13?**
   - ✓ Yes, it is satisfactory but to say depression during pregnancy the pregnant women should have an Edinburgh Postnatal Depression Scale (EPDS) score of 13 and above. It should fulfill the definition of depression during pregnancy.
6. **Please make sure your references are in line with the journal requirements. There are references without authors' names**
   - ✓ We will correct it based on the comment you have provided

Reviewer #3

1. Neglected by whom? by the researcher, clinicians, health planners orr policy maker?

- ✓ Corrected

2. **If you add sample size, the study population, analysis runs to identify the associated factors and the cut-off score**

- ✓ Incorporated

3. **this was in your first manuscript [AOR=0.015(0.001-0.236) So, how come this significant change appeared now?**

- ✓ The original was a type error. We corrected it when we noticed the error.

4. Again.... this is a wide confidence interval. this may be due to inadequate sample size, Data itself is not consistent, or you have outliers in the data, or you have poorly specified model, or you have (partial) collinearity between the variables. The authors should give an explanation

- ✓ We have don collinearity test which showed no significant collinearity and the model was fitted well and again as you saw, we calculated the sample size properly. But small sample size may be the major reason.

5. Significant percentages of your study participants had low levels of educational status. so, how could they know whether their previous diagnosis was depression or other mental health problems.

Again some of your variables were assessed through YES or NO questions. I don't think that assessing this variable with simple YES or NO questions reveal the real problems (e.g. Previous history of depression, Family history of a depressive episode, History of chronic illness)

- ✓ Even those more educated people could not know exactly whether it is depression or not. As we have seen we have used the validated tool to diagnose depression. So based on the tool we diagnosed whether it was depression or not. Again they knew their medical status when they vised the doctor and were proven by the clinician. So they can know their Previous history of depression, Family history of a depressive episode, History of chronic illness from health care providers.

6. how come 17.8% became fifth. Please add nearly or approximately

- ✓ Corrected based on the comment provided

7. This paragraph is just a personal opinion and not fact-based. There are plenty of cross-sectional studies on antenatal depression in Ethiopia, there are also abundant systematic reviews. Please see this links (https://journals.plos.org/plosone/article?id=10.1371/journal.pone.0211764),

(https://bmcpregnancychildbirth.biomedcentral.com/articles/10.1186/s12884-018-2101-x),(Epidemiology of antenatal depression in Africa: a systematic review and meta-analysis), (https://www.hindawi.com/journals/drt/2018/3649269/)
and many more Including all age groups, facilities, and other socio-demographic factors

- ✓ We have taken the Idea and have corrected it.

8. Your study also on women who have ANC follow-up.

- ✓ Noted and corrected

9. In general the organization of the introduction is poor. e.g the gap should be stated at the end of the introduction section. please also revise the introduction section to have a flow for the reader

- ✓ Thanks, we have corrected your concern.

10. The organization and the contents are poor. please revise

- ✓ Thank you. We revised it.

11. The reference is not accessible up on google search

- ✓ Yes, you are correct. We received this information from officials in person.

12. gain the authors should explain the sampling techinique. You have stated stratified sampling as your sampling techniques, so how is all pregnant woman during the study period included in your study?

- ✓ It has been there in the manuscript. All eligible and consenting women attending for antenatal care during the study period were taken into the study consecutively until the sample size was reached.

13. you have said under study participant section all women who visited ANC service were included. here you have selected a small portion of women. how did you select this woman? The sampling section needs major revision? or else the study participants were wrongly sampled.

- ✓ Under the participant section, we have talked about source population, study population, and eligibility criteria. In the sampling section, we have included the participants consecutively until the calculated sample size was reached.

14. what is their profession?

- ✓ midwives

15. who have done the translations?
    - ✓ Language experts

16.What is the findings in pre-test?where was the pre-test site?
- ✓ We did not do analysis but we checked the tool and some corrections were done on the tool. The site was in debre elias woreda

17. where is the reliability of the tools used in your study?
- ✓ Since the tool was validated and confirmed as it was reliable, we didn't do for these tests.

18. add your reference
- ✓ Incorporated.

19. How did you measure the fitness of the model? how did you measure the strength of association?
- ✓ We have used the chi-square fitness model and the strength of the association was measured by the association between dependent and independent variables. (if the confidence interval is low, there will be a strong association.

20. is this a data analysis technique?
- ✓ Corrected

21. Be consistent in one area you have used numbers in another area wording of the number .Please revise this in whole of your document
- ✓ Revised

22. The discussion section lack enough justification based on realistic and scientific evidences for the discrepancies of findings in this study and other studies .please revise
- ✓ We justified based on our findings and the knowledge we have. We have also tried to justify with scientific evidence.

23.the justification for the difference in the prevalence is not based on your findings, realistic and scientific.

Even you have used two Ethiopian studies for comparison. one is higher and another is lower than your finding. this needs justification

- ✓ We justified as this difference might be due to the difference in their population demographic characteristics, study design, period, and the difference in their investigatory or diagnostic tools and we have used Ethiopian studies.

24. This is what is in table 0.48(0.07, 3.21)

- ✓ Sorry for the many editorial and typing errors but thanks to you now we correct them. Pardon.

25 he authors should explain this wide confidence interval?

- ✓ Possibly due to the small sample size

26. This is not your methodological limitation

- ✓ corrected

27. ???

- ✓ since the source was primary, we can take it as the strength of the study.

28. please merge the contents under the conclusion section. Please also follow the journal submission guideline for the contents

- ✓ incorporated

29. our references are not based on the journal referencing style. please revise

- ✓ revised
