# Supplementary material for: Prevalence of depression and associated factors among pregnant women attending antenatal care in public health institutions of Awabale Woreda, East Gojjam Zone, Northwestern Ethiopia: A cross-sectional study
Source: PLoS One. 2022 Oct 13;17(10):e0271876. doi: 10.1371/journal.pone.0271876 (PMC9560138; doi:10.1371/journal.pone.0271876)
Supplement: Attachment — Submitted filename: Response to reviewers.docx [file pone.0271876.s002.docx]

**Dear all reviewers thank you for your constructive comments, here are some responses to the questions raised on your side**

**Reviewer#1**

- We have excluded literature that has a different study population from our study, like [Ayano et al (2019) which deals with about homeless.
- We excluded those who were critically ill during the data collection period. The main reason was they could not give a full response to our questions related to the study.
- We have used stratified random sampling because it is more representative propriety than cluster sampling.
- Even though there were more eligible participants, we have included only 354 respondents due to their preference (that is why we have added a 10% none response rate)
- Thank you for your third comment but we have prepared it based on Plos one journal protocol.
- Even though study instruments are not validated for the specific population, it is better to use them because of no validated tool for a specific population.
- We have explained the associations between dependent and independent variables. The purpose of using binary logistic regression is to explain the association between dependent and independent variables.
- The main reason we used multivariable logistic regression is to manage confounders so it simply explains how we managed the confounding factors. Listing confounding factors is not that much important rather the way of management is mandatory, that is why we have used a multivariable logistic regression model.
- Since we have used the confidence interval and the unadjusted odds value in the table there is no need to put it on the text. Confidence interval is enough and preferred than p-value, so the p-value is not needed. We have put the Bivariable values as "**COR (95%CI)” in the regression table.**
- We have used literature that had a similar study population and single papers related to antenatal depression, we avoided studies done in the homeless and general population.

**Reviewer#2**

- Thank you for the recommendation to re-analysis by using other models, but the model we have used is also scientifically validated. Using the recommended model may not produce a major difference, so to avoid time wastage it will be better to use the EPDS model.

**Reviewer#3**

- Most of the researches was undertake on postnatal depression. The majority of studies on the prevalence of antenatal depression and associated factors have been conducted in developed countries. However, there is a paucity of studies, which examined the prevalence and associated factors of antenatal depression in low-income countries like Ethiopia. There are studies undertaken on antenatal depression. Yet, they focus on adolescent pregnant women and women that have ANC service in hospital settings. Those studies have a limitation on addressing pregnant women in all age groups. In addition, they are likely to have a bias because most women with hindrance in their pregnancies are likely to seek ANC services in hospitals. There are studies, which were excluded illiterate women from the study. This may lead to selection bias because educational status has a significant association with antenatal depression as described in the literature. Factors associated with antenatal depression are not well study in most rural low-income countries like Ethiopia. In a study conducted on the prevalence of antenatal depression and associated factors in Addis Ababa, Ethiopia comorbid illnesses and gender violence were not assessed. This may hinder the result of the finding and the possible association of factors with antenatal depression. The prevalence of antenatal depression also varies in different places. These variations indicate that a study to assess the prevalence and associated factors with antenatal depression in the local context should be undertaken.
- Even though we have used a stratified sampling method to select public health institutions we selected the samples consecutively because they had no sampling frame and difficult to do it with every k^th^ interval (it was in the outpatient setting).
- Adama and awabel may not have different health care services because they are led by similar health care delivery systems ( health care providers, health institutions, and other related services) guided by a similar agency like the ministry of health. The population may have socio-cultural and/or economical differences but we can use it since there is no other study near to awabel other than Adama.
- Since there is no validated tool for antenatal depression, it would be better to use a validated tool for postnatal depression.
- The fitness model was assessed by chi-square test and the strength of the association was measured by adjusted odds ratio.
- Our base to classify rural-urban was based on residence.
- Income was categorized based on a previous study.
- Is there any family history of depressive episodes? Was the question for family history of the depressive episode? They know if the family member has ever experienced sadness or loss of interest in normal activities almost daily for two weeks or more.
- History of chronic illness was asked, Do you have any chronic illness? And they know if they have any history of
  - Diabetes mellitus
  - Hypertension
  - HIV/AIDS
  - Others
