# Supplementary material for: Prevalence of depression and associated factors among pregnant women attending antenatal care in public health institutions of Awabale Woreda, East Gojjam Zone, Northwestern Ethiopia: A cross-sectional study
Source: PLoS One. 2022 Oct 13;17(10):e0271876. doi: 10.1371/journal.pone.0271876 (PMC9560138; doi:10.1371/journal.pone.0271876)
Supplement: Attachment — Submitted filename: response to reviewers.docx [file pone.0271876.s005.docx]

**Responses for reviewers**

**Thank you all for your constructive suggestions and comments.**

**Reviewer #1:**

**1. The main reasons that we did this study were:**

- Most of the researches was undertaking on postnatal depression.
- The majority of studies have been conducted in developed countries.
- There are studies undertaken on antenatal depression. Yet, they focus on adolescent pregnant women and women's that have antenatal care service in hospital settings that couldn’t include in all public health settings.
- Those studies have a limitation on addressing pregnant women in all age groups.
- In addition, they are likely to have a bias because most women with hindrance in their pregnancies are likely to seek antenatal care services in hospitals.
- There are studies, which were excluded illiterate women from the study.
- Federal Democratic Republic of Ethiopia National Mental Health Strategy promotes a decentralized approach in which mental health services are to be offered from local health institutions up to tertiary hospitals to ensure access to treatment near to their living area and with a less restraining setting
  - So, the result of this this study will be important for concerned bodies the see the effectiveness of the strategy and to develop new strategy if needed.

**2. The methods**

- We have revised this section based on the given comment.

**Reviewer #2**

**Even though there is studies done in this area**

- They focus on adolescent pregnant women and women's that have antenatal care service in hospital settings.
- Those studies have a limitation on addressing pregnant women in all age groups.
- In addition, they are likely to have a bias because most women with hindrance in their pregnancies are likely to seek antenatal care services in hospitals.
- There are studies, which were excluded illiterate women from the study.

**Reviewer #3**

Dear reviewer #3, as my view I addressed all of your comments, but some comments were accepted and corrections were done on the manuscript.

- **The main reasons that we did this study were:**
- Most of the researches was undertaking on postnatal depression.
- The majority of studies have been conducted in developed countries.
- There are studies undertaken on antenatal depression. Yet, they focus on adolescent pregnant women and women's that have antenatal care service in hospital settings.
- Those studies have a limitation on addressing pregnant women in all age groups.
- In addition, they are likely to have a bias because most women with hindrance in their pregnancies are likely to seek antenatal care services in hospitals.
- There are studies, which were excluded illiterate women from the study.
- Federal Democratic Republic of Ethiopia National Mental Health Strategy promotes a decentralized approach in which mental health services are to be offered from local health institutions up to tertiary hospitals to ensure access to treatment near to their living area and with a less restraining setting
  - So, the result of this this study will be important for concerned bodies the see the effectiveness of the strategy and to develop new strategy if needed.
- In a study conducted on the prevalence of antenatal depression and associated factors in Addis Ababa, Ethiopia comorbid illnesses and gender violence were not assessed. This may hinder the result of the finding and the possible association of factors with antenatal depression. The prevalence of antenatal depression also varies in different places. These variations indicate that a study to assess the prevalence and associated factors with antenatal depression in the local context should be undertaken.
- Even though we have used a stratified sampling method to select public health institutions we selected the samples consecutively because they had no sampling frame and difficult to do it with every k^th^ interval (it was in the outpatient setting).
- Adama and awabel may not have different health care services because they are led by similar health care delivery systems ( health care providers, health institutions, and other related services) guided by a similar agency like the ministry of health. The population may have socio-cultural and/or economical differences but we can use it since there is no other study near to awabel other than Adama.
- Since there is no validated tool for antenatal depression and the study populations were mothers, it would be better to use a validated tool than simple tool
- The fitness model was assessed by chi-square test and the strength of the association was measured by adjusted odds ratio.
- Our base to classify rural-urban was based on residence.
- Income was categorized based on a previous study.
- Is there any family history of depressive episodes? Was the question for family history of the depressive episode? They know if the family member has ever experienced sadness or loss of interest in normal activities almost daily for two weeks or more.
- History of chronic illness was asked, Do you have any chronic illness? And they know if they have any history of
  - Diabetes mellitus
  - Hypertension
  - HIV/AIDS
  - Others
