# Supplementary material for: Prevalence of depression and associated factors among pregnant women attending antenatal care in public health institutions of Awabale Woreda, East Gojjam Zone, Northwestern Ethiopia: A cross-sectional study
Source: PLoS One. 2022 Oct 13;17(10):e0271876. doi: 10.1371/journal.pone.0271876 (PMC9560138; doi:10.1371/journal.pone.0271876)
Supplement: Attachment — Submitted filename: response to reviewers.docx [file pone.0271876.s007.docx]

***Dear reviewers***

***Thank you for your positive and constructive comments. I have provided responses based on your comments and questions.***

**Reviewer #1**

# 1. There is a paucity of data on antenatal depression in Ethiopia, yet there is a lot of work published from 2006 (Hanlon et al) to 2021.

# We have corrected it based on the given comment

2. **Can you please acknowledge the source of this information - REFERENCES?**

# This is our suggestion we have corrected it.

# 3. What is the difference between your study site/population and that which has been reported in the literature already?

# The findings in the previous study were not consistent which shows further study was needed

# The study population of our study area is different from other study populations like Adama and Addis Ababa and other places through lifestyle and perception about pregnancy and its importance.

4. **Ethically, women aged below 18 cannot consent. How did you cater to them?**

# We took assent from their family if they came with them. If they came alone, we took assent from their health care providers

5. **The EPDS was validated in Ethiopia and a cut-off score of 7/8 was found to be satisfactory. Then why did you use a cut-off score of 13?**
   - Yes, it is satisfactory but to say depression during pregnancy the pregnant women should have an Edinburgh Postnatal Depression Scale (EPDS) score of 13 and above. It should fulfill the definition of depression during pregnancy.
6. **Please make sure your references are in line with the journal requirements. There are references without authors' names**
   - We will correct it based on the comment you have provided

**Reviewer #3**

**1. Neglected by whom? by the researcher, clinicians, health planners orr policy maker?**

- Corrected

2. **If you add sample size, the study population, analysis runs to identify the associated factors and the cut-off score**

- Incorporated

3. **this was in your first manuscript [AOR=0.015(0.001-0.236) So, how come this significant change appeared now?**

- The original was a type error. We corrected it when we noticed the error.

**4. Again.... this is a wide confidence interval. this may be due to inadequate sample size, Data itself is not consistent, or you have outliers in the data, or you have poorly specified model, or you have (partial) collinearity between the variables. The authors should give an explanation**

- We have don collinearity test which showed no significant collinearity and the model was fitted well and again as you saw, we calculated the sample size properly. But small sample size may be the major reason.

5. Significant percentages of your study participants had low levels of educational status. so, how could they know whether their previous diagnosis was depression or other mental health problems.

Again some of your variables were assessed through YES or NO questions. I don't think that assessing this variable with simple YES or NO questions reveal the real problems (e.g. Previous history of depression, Family history of a depressive episode, History of chronic illness)

- Even those more educated people could not know exactly whether it is depression or not. As we have seen we have used the validated tool to diagnose depression. So based on the tool we diagnosed whether it was depression or not. Again they knew their medical status when they vised the doctor and were proven by the clinician. So they can know their Previous history of depression, Family history of a depressive episode, History of chronic illness from health care providers.

6. how come 17.8% became fifth. Please add nearly or approximately

- Corrected based on the comment provided

7. This paragraph is just a personal opinion and not fact-based. There are plenty of cross-sectional studies on antenatal depression in Ethiopia, there are also abundant systematic reviews. Please see this links (https://journals.plos.org/plosone/article?id=10.1371/journal.pone.0211764), (https://bmcpregnancychildbirth.biomedcentral.com/articles/10.1186/s12884-018-2101-x),( Epidemiology of antenatal depression in Africa: a systematic review and meta-analysis), (https://www.hindawi.com/journals/drt/2018/3649269/)

and many more Including all age groups, facilities, and other socio-demographic factors

- We have taken the Idea and have corrected it.

8. Your study also on women who have ANC follow-up.

- Noted and corrected

9. In general the organization of the introduction is poor. e.g the gap should be stated at the end of the introduction section. please also revise the introduction section to have a flow for the reader

- Thanks, we have corrected your concern.

10. The organization and the contents are poor. please revise

- Thank you. We revised it.

11. The reference is not accessible up on google search

- Yes, you are correct. We received this information from officials in person.

12. gain the authors should explain the sampling techinique. You have stated stratified sampling as your sampling techniques, so how is all pregnant woman during the study period included in your study?

- It has been there in the manuscript. All eligible and consenting women attending for antenatal care during the study period were taken into the study consecutively until the sample size was reached.

13. you have said under study participant section all women who visited ANC service were included. here you have selected a small portion of women. how did you select this woman? The sampling section needs major revision? or else the study participants were wrongly sampled.

- Under the participant section, we have talked about source population, study population, and eligibility criteria. In the sampling section, we have included the participants consecutively until the calculated sample size was reached.

14. what is their profession?

- midwives

15. who have done the translations?

- Language experts

16.What is the findings in pre-test?where was the pre-test site?

- We did not do analysis but we checked the tool and some corrections were done on the tool. The site was in debre elias woreda

17. where is the reliability of the tools used in your study?

- Since the tool was validated and confirmed as it was reliable, we didn’t do for these tests.

18. add your reference

- Incorporated.

19. How did you measure the fitness of the model? how did you measure the strength of association?

- We have used the chi-square fitness model and the strength of the association was measured by the association between dependent and independent variables. (if the confidence interval is low, there will be a strong association.

20. is this a data analysis technique?

- Corrected

21. Be consistent in one area you have used numbers in another area wording of the number .Please revise this in whole of your document

- Revised

22. The discussion section lack enough justification based on realistic and scientific evidences for the discrepancies of findings in this study and other studies .please revise

- We justified based on our findings and the knowledge we have. We have also tried to justify with scientific evidence.

23.the justification for the difference in the prevalence is not based on your findings, realistic and scientific.

Even you have used two Ethiopian studies for comparison. one is higher and another is lower than your finding. this needs justification

- We justified as this difference might be due to the difference in their population

demographic characteristics, study design, period, and the difference in their investigatory or diagnostic tools and we have used Ethiopian studies.

24. This is what is in table 0.48(0.07, 3.21)

- Sorry for the many editorial and typing errors but thanks to you now we correct them. Pardon.

25 he authors should explain this wide confidence interval?

- Possibly due to the small sample size

26. This is not your methodological limitation

- corrected

27. ???

- since the source was primary, we can take it as the strength of the study.

28. please merge the contents under the conclusion section. Please also follow the journal submission guideline for the contents

- incorporated

29. our references are not based on the journal referencing style. please revise

- revised
