# Supplementary material for: Prevalence of depression and associated factors among pregnant women attending antenatal care in public health institutions of Awabale Woreda, East Gojjam Zone, Northwestern Ethiopia: A cross-sectional study
Source: PLoS One. 2022 Oct 13;17(10):e0271876. doi: 10.1371/journal.pone.0271876 (PMC9560138; doi:10.1371/journal.pone.0271876)
Supplement: Attachment — Submitted filename: response to reviewers.docx [file pone.0271876.s009.docx]

Dear reviewer #3 thank you for your constructive comments and suggestions. Here are some responses to raised issues.

1. I still has concern on wide confidence interval. the authors should explain this "

- *Like other variables, [AOR18.15 (2.73-120.76)] is gained from the analysis process of the data. This may be due to different reasons like small sample size or inappropriate responses of the study participants*

*2.* Significant percentages of your study participants had low levels of educational status. so, how could they know whether their previous diagnosis was depression or other mental health problems. this is ambiguous unless the medical record of the study respondents reviewed

- *They got the information from their health care providers who diagnosed them.*

3. if all pregnant women in the study period included in your study, you have used consecutive sampling technique which is not a probability sampling technique. in that sense it is difficult to generalize your finding. I expect randomization for generalize

- We were talking about the study population here. But we can use consecutive sampling techniques in special cases like busy work areas.

4. I really don't see the importance of this.

- The Federal Democratic Republic of Ethiopia's National Mental Health Strategy promotes a decentralized approach in mental health services from local health institutions up to tertiary hospitals but service is not being given as expected. So this is important to get brief information about the prevalence and factors of depression during pregnancy.

5. Please put the reliability of EPDS and Oslo social support scales in your study (cronbatch alpha result)

- This tool is a validated tool so putting the reliability of the validated tool may not have a value.

6. better to mention some variables were assessed using yes or no response which might not reflect the.....

- Since we asked yes/no questions, we are not sure they were giving true (diagnosed responses), so we cannot mention the variables that were not confirmed
