# Supplementary material for: Prevalence of depression and associated factors among pregnant women attending antenatal care in public health institutions of Awabale Woreda, East Gojjam Zone, Northwestern Ethiopia: A cross-sectional study
Source: PLoS One. 2022 Oct 13;17(10):e0271876. doi: 10.1371/journal.pone.0271876 (PMC9560138; doi:10.1371/journal.pone.0271876)
Supplement: Attachment — Submitted filename: Response to the reviewer.docx [file pone.0271876.s010.docx]

**Response to the reviewer**

**I would like to forward my heart felt gratitude for your comments and time you spent on this paper. Here are point by point responses for your questions you raised.**

1. Please explain what your study is adding to the body of knowledge.

- **The studies you mentioned as they were done in Ethiopia were done before 11 years so that there may be possible population and environmental change. Again even though there are some current studies in Ethiopia, no study done in study done in the study area which is different in economic and socio-cultural aspect.**

2. Please refer to such studies for your introduction to be more convincing

- **Corrected**

3. Can they please revise their limitations?

- **Corrected**

4. Can the authors please detail how they came up with their sample size?

- **Corrected**
  - The sample size was calculated based on a single population proportion formula assumption. The prevalence of Antenatal depression was 31.2 % from a study conducted in in Adama, Ethiopia [15] and a 5% margin of error was used. $initialsamplesize ={(Z\frac{a}{2})}^{2}*\frac{p(1-p)}{w2}={1.96}^{2}*\frac{0.312\left( 1-0.312 \right)}{({0.05)}^{2}}=330$. We have added a non-response rate of 10% and 330*0.10=33. Then the final sample size was 330+33=363.

5. EPDS is a screening tool hence the pregnant women will have depressive symptoms, not depression.
   The authors should revise their statement “About 63 (17.8%) had antenatal depression.”

- **Corrected**

6. What do the authors mean by “Two hundred nine (59%) of respondents had a history of pregnancy”? Is it that they were multipara? Please use the correct medical terminology.

- **Corrected**

7. Bare of the statistics

- **Corrected**
