# Supplementary material for: Prevalence of depression and associated factors among pregnant women attending antenatal care in public health institutions of Awabale Woreda, East Gojjam Zone, Northwestern Ethiopia: A cross-sectional study
Source: PLoS One. 2022 Oct 13;17(10):e0271876. doi: 10.1371/journal.pone.0271876 (PMC9560138; doi:10.1371/journal.pone.0271876)
Supplement: Attachment — Submitted filename: Response to reviewers.docx [file pone.0271876.s011.docx]

**Response to reviewers**

Dear reviewers, thank you for your time you spent on this manuscript and your constructive comments you provided. Here below, we have provided response to your question.

1. We have checked the references and we got an error on reference number 8 and 19.

2. Based on your suggestion we corrected the reference number 8, but the reference number 19 is the annual report of the district which is not published. so, it will be better if you use as a direct reference.
